# An unexpected role for the ketogenic diet in triggering tumor metastasis by modulating BACH1-mediated transcription

**DOI:** 10.1126/sciadv.adm9481

**Published:** 2024-06-05

**Authors:** Zhenyi Su, Yanqing Liu, Zhangchuan Xia, Anil K. Rustgi, Wei Gu

**Affiliations:** ^1^ Institute for Cancer Genetics, and Department of Pathology and Cell Biology, Vagelos College of Physicians and Surgeons, Columbia University, 1130 Nicholas Ave, New York, NY 10032, USA.; ^2^ Herbert Irving Comprehensive Cancer Center, Vagelos College of Physicians and Surgeons, Columbia University, 1130 Nicholas Ave, New York, NY 10032, USA.; ^3^ Division of Digestive and Liver Diseases, Department of Medicine, Herbert Irving Comprehensive Cancer Center Vagelos College of Physicians and Surgeons, Columbia University Irving Medical Center, New York, NY 10032 USA.

## Abstract

We have found that the ketogenic (Keto) diet is able to, unexpectedly, promote the metastatic potential of cancer cells in complementary mouse models. Notably, the Keto diet–induced tumor metastasis is dependent on BTB domain and CNC homolog 1 (BACH1) and its up-regulation of pro-metastatic targets, including cell migration–inducing hyaluronidase 1, in response to the Keto diet. By contrast, upon genetic knockout or pharmacological inhibition of endogenous BACH1, the Keto diet–mediated activation of those targets is largely diminished, and the effects on tumor metastasis are completely abolished. Mechanistically, upon administration of the Keto diet, the levels of activating transcription factor 4 (ATF4) are markedly induced. Through direct interaction with BACH1, ATF4 is recruited to those pro-metastatic target promoters and enhances BACH1-mediated transcriptional activation. Together, these data implicate a distinct transcription regulatory program of BACH1 for tumor metastasis induced by the Keto diet. Our study also raises a potential health risk of the Keto diet in human patients with cancer.

## Teaser

BACH1 is critical for tumor metastasis induced by the Keto diet.

## INTRODUCTION

Breast cancer remains one of the leading causes of cancer-related deaths among women worldwide (*1*, *2*). Over time, research has provided valuable insights into the genetic, molecular, and environmental factors that contribute to breast cancer progression and metastasis (*3*–*5*). One area that has piqued interest is the relationship between nutrient availability and tumor behavior. The ketogenic (Keto) diet, characterized by low carbohydrate, high fat, and adequate protein intake, has been reported to have a notable impact on weight loss, epilepsy, diabetes, cardiovascular health, and cancer (*6*–*9*). The Keto diet is increasingly being considered as an adjuvant approach in cancer therapy due to its observed effects on slowing tumor growth (*10*–*12*). Mechanistically, the Keto diet counteracts the Warburg effect, where cancer cells primarily depend upon glycolysis for adenosine 5′-triphosphate production rather than oxidative phosphorylation. Implementation of this diet diminishes glucose availability, thereby causing cancer cells to be deprived of energy. In addition, some cancer types exhibit an inability to process ketone bodies. Moreover, by decreasing glucose levels, there is a concurrent reduction in insulin and insulin-like growth factor levels, both of which may accelerate cancer cell growth (*13*). More recently, ferroptosis has been proposed to be a contributor to the Keto diet–mediated antitumor effect (*14*). However, while the impact of the Keto diet on primary tumors is relatively well-documented, its effects on metastatic progression, a critical cause of cancer-related mortality, remain unclear (*15*).

BTB domain and CNC homolog 1 (BACH1), a heme-binding protein from the basic leucine zipper factor family, plays multifaceted roles in oxidative stress regulation, tumor metastasis, and mitochondrial metabolism in breast cancer (*16*–*20*). As a DNA-binding transcription factor, BACH1 forms heterodimers with small MAF proteins (*21*). This interaction allows BACH1 to suppress a series of anti-oxidative genes, such as solute carrier family 7 member 11 (*SLC7A11*) and heme oxygenase 1 (*HMOX1*), while simultaneously activating genes involved in tumor metastasis, including matrix metallopeptidase 1 (*MMP1*), C-X-C motif chemokine receptor 4 (*CXCR4*), *IL-11*, and hexokinase 2 (*HK2*) (*18*, *22*–*25*). A recent study by our group revealed that BACH1 mediates the pro-metastatic activity of hotspot p53 mutant p53^R175H^ and identified cell migration–inducing hyaluronidase 1 (*CEMIP*) as a BACH1–up-regulated gene that is crucial for metastasis (*26*).

In this study, we used a carbohydrate-free Keto diet and observed the dual role of Keto diet in breast cancer. On the one hand, the Keto diet suppresses primary tumor growth, but, on the other hand, it simultaneously enhances the metastatic capacity of breast cancer cells. Intriguingly, the Keto diet–induced metastasis is abrogated by BACH1 depletion. We next explored the mechanisms by which BACH1 connects with Keto diet–induced metastasis. By performing RNA sequencing (RNA-seq) analysis, we detected an up-regulation in BACH1-modulated pro-metastatic genes, including *CEMIP*, upon glucose starvation. Mechanistically, we found an interaction between BACH1 and ATF4, a protein whose levels markedly increase upon glucose starvation or Keto diet. This interaction intensifies the DNA binding activity of BACH1 to its target promoters, thereby amplifying BACH1-mediated transcription of pro-metastatic genes.

## RESULTS

### Keto diet suppresses primary tumor growth but paradoxically promotes tumor metastasis

**Fig. 1.**
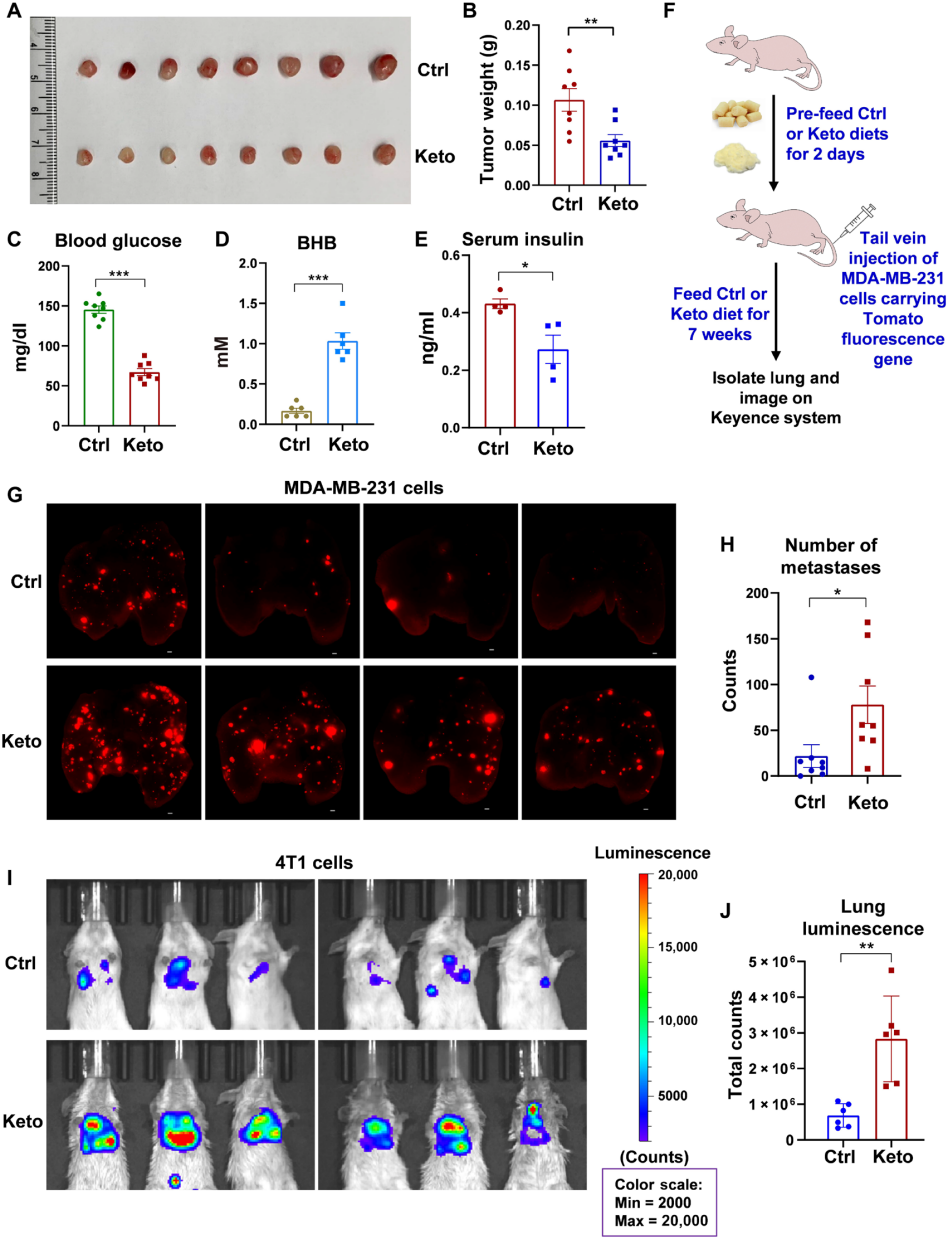
Keto diet reduces primary tumor growth but enhances tumor metastasis. (**A**) MDA-MB-231 cell xenograft tumors isolated from nude mice fed either a control (Ctrl) diet or a Keto diet. (**B**) Tumor weights. *n* = 8 mice for each group. (**C**) Quantitation of blood glucose levels after placing mice on Ctrl or Keto diet for 4 weeks. Mouse blood glucose levels were determined by OneTouch Verio Reflect meter (LifeScan). *n* = 8 mice for each group. (**D**) Blood β-hydroxybutyrate (BHB) levels after feeding with Ctrl or Keto diet for 2 weeks. *n* = 6 mice. (**E**) Serum insulin levels after feeding with Ctrl or Keto diet for 2 weeks. *n* = 4 mice. (**F**) Diagram for metastasis model and mouse feeding regimen. (**G**) Representative fluorescence images of the lungs infiltrated with MDA-MB-231 tumors. Each red spot represents a metastatic nodule. Scale bars, 500 μm. (**H**) Quantitative analysis of number of metastases in lungs using BZ-X800 Analyzer software, related to (G). *n* = 8 mice for each group. (**I**) In vivo luminescence imaging of BALB/c mice injected with luciferase-expressing 4T1 cells via the tail vein. Mice began receiving either the Ctrl or Keto diet 2 days before the tumor cell injection. For in vivo imaging, mice were intraperitoneally injected with luciferase substrate d-luciferin and imaged on IVIS Spectrum Optical Imaging System. *n* = 6 mice for each group. (**J**) Quantitative analysis of total counts of luminescence in lungs, related to (I). *n* = 6 mice for each group. In (B), (C) to (E), (H), and (J), data represent means ± SEM; *P* values were calculated using unpaired two-tailed Student’s *t* test. *P < 0.05, **P < 0.01, and ***P < 0.001.

To investigate the impact of Keto diet on breast tumor behavior, we first established a xenograft model using MDA-MB-231 breast cancer cells to examine primary tumor growth. We used a carbohydrate-free Keto diet with a ratio of fat to protein 4.3:1 by weight (see formula in table S1). Consistent with previous studies, mice on a Keto diet, in contrast to those on a control (Ctrl) diet, displayed a noticeable reduction in primary tumor growth (Fig. 1, A and B). Blood glucose levels significantly decreased under the Keto diet treatment (Fig. 1C). Furthermore, blood ketone levels significantly increased after 2 weeks on the Keto diet (Fig. 1D), suggesting that the animals were effectively generating ketone bodies upon glucose deprivation. As shown in Fig. 1E, serum insulin levels in mice significantly decreased after 2 weeks of Keto diet feeding. Long-term consumption of Keto diet reduces insulin levels, which may reduce insulin–insulin-like growth factor 1 receptor–mediated tumor growth and progression (*27*).

Unexpectedly, while primary tumor growth was hampered, tumor metastasis was enhanced in mice fed with a Keto diet. In a mouse tail vein injection model using MDA-MB-231 cells carrying a tomato fluorescence gene, we observed a notable increase in the number of metastatic nodules in the lungs of mice on the Keto diet, compared to mice on the Ctrl diet (Fig. 1, F to H).

To validate further these findings, an independent experiment using the luciferase-expressing mouse breast cancer cell line 4T1 was undertaken. 4T1 cells were injected into BALB/c mice via the tail vein, and metastatic spread was imaged on the IVIS Spectrum Optical Imaging System (Fig. 1I). As shown in Fig. 1 (I and J), more lung metastases were observed in the Keto diet group, compared to the Ctrl diet group.

Next, we further investigated the impact of Keto diet on metastasis using a mammary fat pad injection model (fig. S1A). BALB/c mice inoculated with luciferase-expressing 4T1 cells were subsequently transitioned to either Ctrl or Keto diet. Similarly, the blood glucose level markedly reduced, while blood ketone levels increased after 2 weeks on the Keto diet (fig. S1, D and E). Three to 3 weeks after dietary intervention, in vivo imaging was conducted to monitor lung metastasis. Notably, under the Keto diet condition, the primary tumor growth was significantly decreased (fig. S1, B and C), whereas the lung metastasis potential was increased, as observed by both in vivo imaging and hematoxylin and eosin (H&E) staining (fig. S1, F to I). Together, these findings reveal a paradoxical role of Keto diet in breast tumor progression: While primary tumor growth is suppressed, metastatic potential is concurrently amplified.

### Keto diet promotes tumor metastasis in a BACH1-dependent manner

Previous research has linked BACH1 to enhanced metastatic potential across a variety of cancers, including breast and lung cancers (*18*, *22*, *28*). Moreover, our recent study demonstrated that BACH1 mediates the pro-metastatic capacity of p53-R175H (*26*). Against this background, we sought to understand the potential relationship between BACH1 and the observed metastatic effects under a Keto diet.

**Fig. 2.**
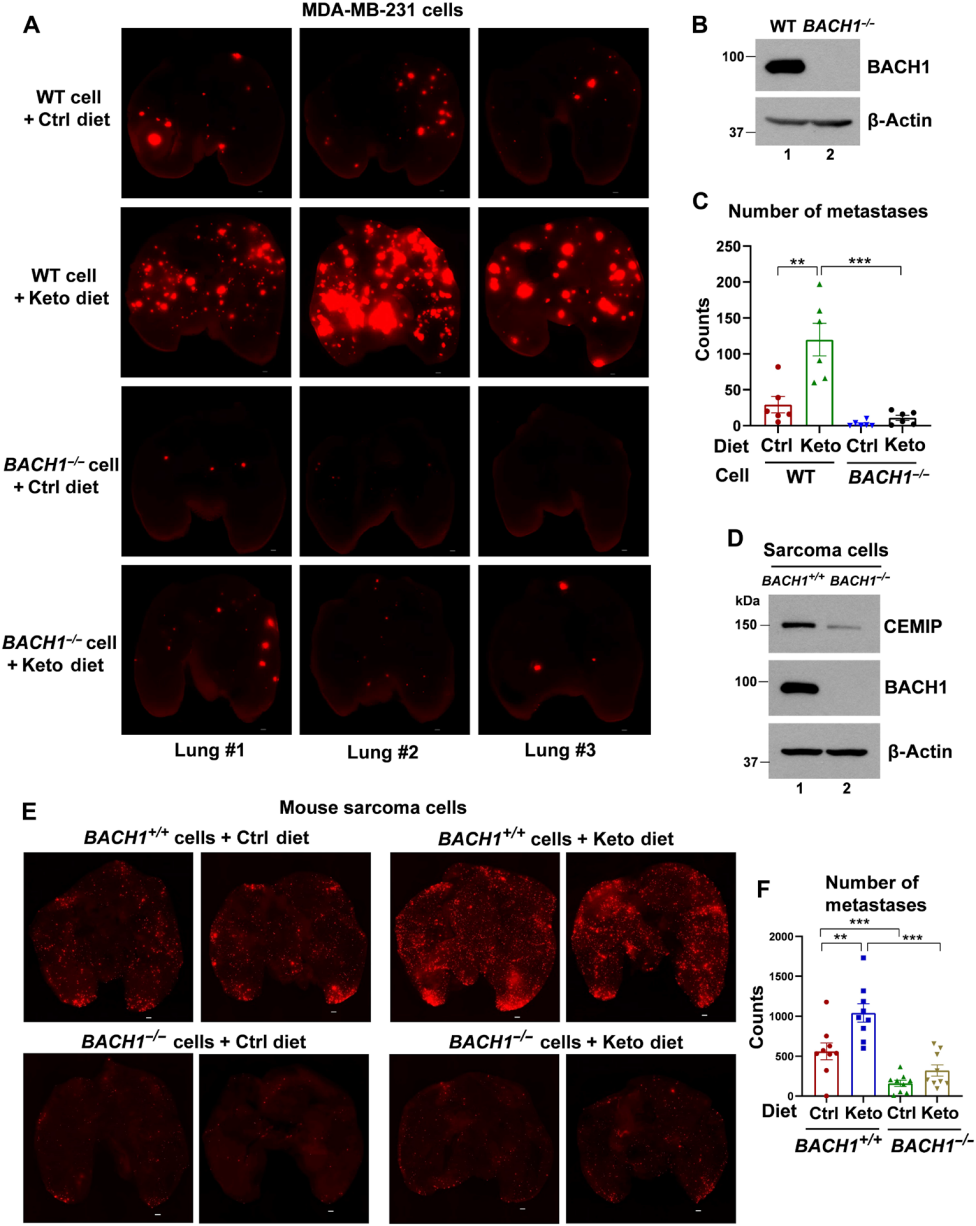
Keto diet–induced tumor metastasis is BACH1-dependent. (**A**) Representative fluorescence images of the lungs infiltrated with MDA-MB-231 WT or *BACH1^−/−^* cells carrying the tomato fluorescence gene. Nude mice were placed on either the Ctrl or Keto diet 2 days before the tail vein injection of tumor cells and were euthanized in the seventh week for imaging. Scale bars, 500 μm. (**B**) Identification of MDA-MB-231 WT or *BACH1^−/−^* cells by Western blot analysis. (**C**) Quantitative analysis of the number of metastases in the lungs using BZ-X800 Analyzer software, related to (A). *n* = 6 mice for each group. (**D**) Identification of BACH1 knockout mouse sarcoma cells by Western blot analysis. (**E**) Representative fluorescence images of the lungs infiltrated with mouse *BACH1^+/+^* or *BACH1^−/−^* sarcoma cells carrying the tomato fluorescence gene. Nude mice were placed on either the Ctrl or Keto diet 2 days before the tail vein injection of tumor cells and were euthanized in the sixth week for imaging. Scale bars, 500 μm. (**F**) Quantitative analysis of the number of metastases in the lungs, as related to (F), using BZ-X800 Analyzer software. *n* = 9 mice for each group. In (C) and (F), data represent means ± SEM; *P* values were calculated using unpaired two-tailed Student’s *t* test. **P < 0.01 and ***P < 0.001

To that end, we generated MDA-MB-231 wild type (WT) or *BACH1^−/−^* cells using the Crispr method, injected them into athymic nude mice via tail vein, and studied the impact of different diets on tumor metastasis. Fluorescence imaging of the lungs, following implantation of MDA-MB-231 WT or *BACH1^−/−^* tumor cells, revealed distinct metastatic patterns in response to dietary shifts. Once again, mice in the Keto diet group exhibited significantly more lung metastases than those in the Ctrl diet group (Fig. 2, A to C). Notably, *BACH1^−/−^* breast cancer cells had significantly reduced metastatic potential in Ctrl diet groups, which is consistent with previous studies showing BACH1 is a pro-metastatic protein (*18*, *22*). BACH1 knockout largely abolished the pro-metastatic potential of MDA-MB-231 cells induced by the Keto diet (Fig. 2, A to C).

We further validated the BACH1 dependency in Keto diet–induced tumor metastasis using a mouse sarcoma model. Fluorescence imaging, followed by quantitative analysis, confirmed the reduced metastatic potential of *BACH1^−/−^* cells, especially when subjected to the Keto diet (Fig. 2, D to F). Together, these findings underscore the pivotal role of BACH1 in mediating the Keto diet–induced metastatic effects.

### Glucose deprivation and Keto diet mimics in vitro up-regulate the expression of BACH1-induced pro-metastatic genes

**Fig. 3.**
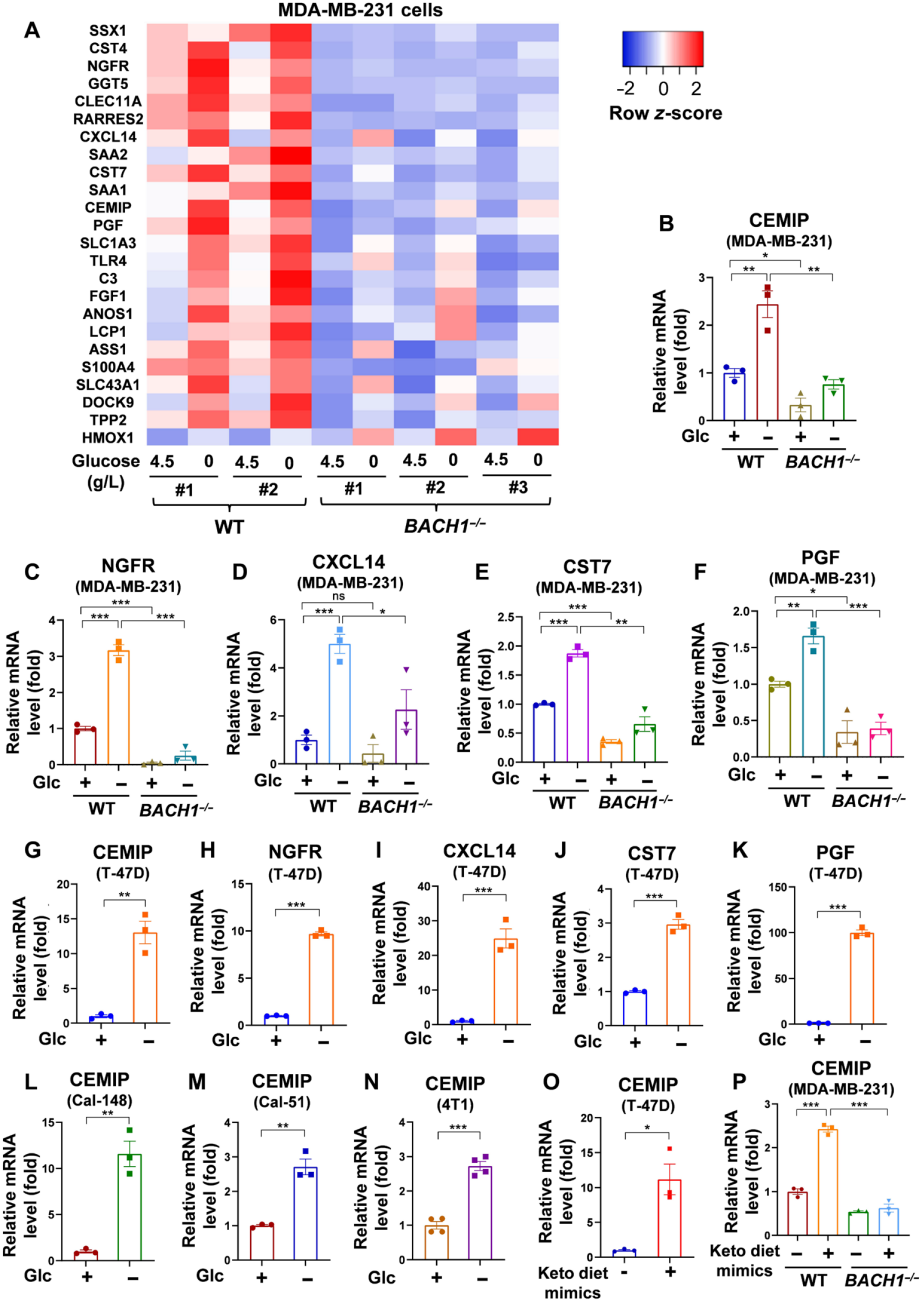
RNA-seq analysis of BACH1–up-regulated pro-metastatic genes upon glucose starvation. (**A**) Heatmap displays the expression of BACH1–up-regulated pro-metastatic targets under both glucose starvation and non-starvation conditions. MDA-MB-231 Ctrl cells (two individual clones) and *BACH1^−/−^* cells (three individual clones) were incubated in DMEM medium with glucose (either 4.5 or 0 g/liter) for 40 hours. Subsequently, the cells were collected for RNA-seq. (**B** to **F**) qPCR validation of several candidates identified from the RNA-seq results of MDA-MB-231 cells. CEMIP (B), NGFR (C), CXCL14 (D), CST7 (E), and PGF (F). (*n* = 3). Glc, glucose. (**G** to **K**) qPCR analysis of the mRNA expression level of CEMIP (G), NGFR (H), CXCL14 (I), CST7 (J), and PGF (K) in T47D cells incubated with glucose (4.5 or 0 g/liter) for 48 hours (*n* = 3). (**L** to **N**) qPCR analysis of CEMIP mRNA expression level in Cal-148, Cal-51, and 4T1 cells incubated with glucose (4.5 or 0 g/liter) for 72, 48, and 60 hours, respectively (L and M, *n* = 3; N, *n* = 4). (**O**) qPCR analysis of CEMIP mRNA expression levels in T47D cells incubated with or without Keto diet mimics in vitro [glucose (0.5 g/liter) + 5 mM β-hydroxybutyrate + 100 μM decanoic acid] for 4 days. *n* = 3. (**P**) qPCR analysis of CEMIP mRNA expression level in MDA-MB-231 WT or *BACH1^−/−^* cells incubated with or without Keto diet mimics in vitro [glucose (0.5 g/liter) + 5 mM β-hydroxybutyrate + 100 μM decanoic acid] in fetal bovine serum–reduced DMEM medium for 4 days. *n* = 3. In (B) to (P), data represent means ± SEM; *n* = 3 or 4 technical replicates; *P* values were calculated using unpaired two-tailed Student’s *t* test. ns, not significant; *P < 0.05, **P < 0.01, and ***P < 0.001.

Because the primary effect of the Keto diet is to establish a glucose-deprived condition in vivo, we first used glucose starvation in vitro to study the effects of the Keto diet. In our endeavor to comprehend the influence of glucose starvation on pro-metastatic genes associated with BACH1, we performed RNA-seq on MDA-MB-231 cells. A heatmap visualizing these data unveiled that many BACH1–up-regulated pro-metastatic targets were also induced under glucose deprivation conditions. When MDA-MB-231 Ctrl cells and distinct *BACH1^−/−^* clones were subjected to either glucose-rich (4.5 g/liter) or glucose-free conditions for 40 hours, we observed a marked induction of these BACH1-responsive targets in glucose-deprived settings (Fig. 3A).

To validate the RNA-seq findings, we used quantitative polymerase chain reaction (qPCR) for analysis of several candidate genes identified from our sequencing results. Notably, pro-metastatic genes such as *CEMIP* (*26*, *29*, *30*), C-X-C motif chemokine ligand 14 (*CXCL14*) (*31*, *32*), nerve growth factor receptor (*NGFR*) (*33*, *34*), cystatin F (*CST7*) (*35*, *36*), and placental growth factor (*PGF*) (*37*) are up-regulated under glucose starvation (Fig. 3, B to F). This up-regulation, however, was counteracted with *BACH1* knockout (Fig. 3, B to F), suggesting BACH1’s pivotal role in modulating these genes’ expressions under glucose-deficient conditions. We further extended our analysis to other breast cancer cell lines, T47D and Cal-148. Consistently, genes such as *CEMIP*, *NGFR*, *CXCL14*, *CST7*, and *PGF* displayed an up-regulation in response to glucose starvation (Fig. 3, G to K, and fig. S2, A to D). Subsequent qPCR evaluations in multiple breast cell lines—Cal-148, Cal-51, and 4T1—after glucose starvation consistently showed the increased expression of CEMIP (Fig. 3, L to N). Our interest particularly focused on CEMIP due to our previous findings, highlighting its pivotal role in BACH1-mediated pro-metastatic functions (*26*). CEMIP has been shown to be a critical pro-metastatic protein in several types of tumors, including breast cancer (*29*, *30*, *38*–*40*). The expression of CEMIP is significantly higher in breast cancer tissues compared to normal breast tissues, and its levels inversely correlate with both overall survival and post-progression survival of patients with breast cancer (*40*).

Glucose deprivation alone may not be sufficient to mimic the Keto diet’s effect in vitro. Next, we used a combined formula [glucose (0.5 to 1 g/liter) + 5 mM β-hydroxybutyrate + 100 to 250 μM decanoic acid] to mimic the Keto diet in vitro (*41*). Similar to the results of glucose starvation alone, the Keto diet mimic treatment significantly increased the expression of pro-metastatic genes, including *CEMIP*, *CXCL14*, *NGFR*, and *CST7* (Fig. 3, O and P, and fig. S2, E to J).

**Fig. 4.**
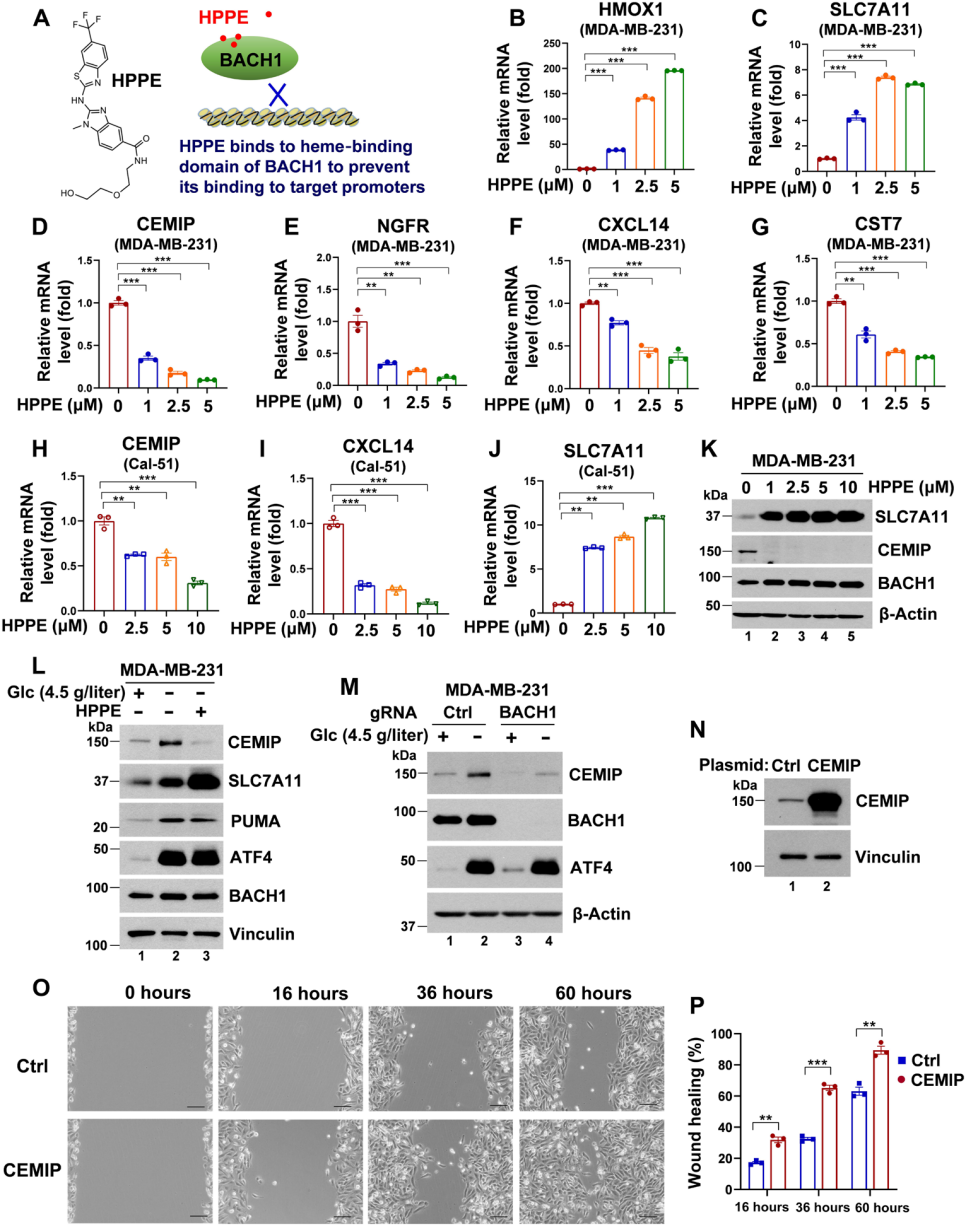
The BACH1 inhibitor, HPPE, suppresses the expression of a number of pro-metastatic genes induced in glucose deprivation. (**A**) Structure and working mechanism of BACH1 inhibitor HPPE. (**B** to **G**) HPPE up-regulates HMOX1 (B) and SLC7A11 (C) mRNA expression but down-regulates CEMIP (D), NGFR (E), CXCL14 (F), and CST7 (G) mRNA expression in MDA-MB-231 cells (*n* = 3). (**H** to **J**) HPPE down-regulates CEMIP and CXCL14 mRNA expression but up-regulates SLC7A11 mRNA expression in Cal-51 cells (*n* = 3). (**K**) Western blot analysis of CEMIP, SLC7A11, and BACH1 expression in MDA-MB-231 cells treated with 0 to 10 μM HPPE for 48 hours. (**L**) Western blot analysis of CEMIP, SLC7A11, PUMA, ATF4, and BACH1 expression in MDA-MB-231 cells pretreated with 2.5 μM HPPE overnight, followed by incubation with glucose DMEM (4.5 or 0 g/liter) for 32 hours. (**M**) Western blot analysis of CEMIP, BACH1, and ATF4 expression in MDA-MB-231 Ctrl or BACH1 Crispr pool cells incubated with glucose DMEM (4.5 or 0 g/liter) for 32 hours. (**N** to **P**) Wound healing assay conducted on MDA-MB-231 cells expressing either Ctrl or CEMIP plasmid across various time points (16 to 60 hours). (N) CEMIP expression as identified by Western blot; (O) representative images of wound healing; and (P) a quantitative analysis of the percentage of wound healing (*n* = 3). In (B) to (J) and (P), data represent means ± SEM; *n* = 3 technical replicates; *P* values were calculated using unpaired two-tailed Student’s *t* test. **P < 0.01 and ***P < 0.001.

To validate further the BACH1 dependency in glucose starvation–induced pro-metastatic gene expression, we used a recently reported BACH1 inhibitor, HPPE (*42*). The mechanism of HPPE involves binding to the heme-binding domain of BACH1, which, in turn, prevents BACH1 from binding to the promoters of target genes (Fig. 4A) (*42*). Upon treating MDA-MB-231 cells with HPPE, the expression of well-known BACH1-suppressive targets, including HMOX1 and SLC7A11, is markedly up-regulated (Fig. 4, B and C). This confirms that HPPE acts as a specific and potent BACH1 inhibitor. Conversely, HPPE reduces the mRNA expression levels of CEMIP, NGFR, CXCL14, and CST7 in a dose-dependent manner, further indicating that these genes are BACH1 up-regulated targets (Fig. 4, D to G). Similar to the effect of HPPE, hemin, a classic BACH1 inhibitor, also reduced the expression of CEMIP, NGFR, and CXCL14, while it up-regulated SLC7A11 expression in a dose-dependent manner, albeit not as strongly as HPPE (fig. S3, A to D). We subsequently validated the effect of HPPE in Cal-51 cells, demonstrating that HPPE suppresses the expression of CEMIP and CXCL14 while enhancing the expression of SLC7A11 (Fig. 4, H to J). Consistent with mRNA data, Western blot analysis of MDA-MB-231 cells showed a marked decrease in CEMIP and a pronounced increase in SLC7A11 expression following HPPE treatment (Fig. 4K). Pretreatment with HPPE inhibited the up-regulation of CEMIP expression induced by glucose starvation (Fig. 4L and fig. S3E). Using BACH1 Crispr pool cells, we further confirmed that glucose deprivation–induced CEMIP expression is BACH1-dependent (Fig. 4M).

Next, we investigated whether an elevated level of CEMIP is associated with increased cell migration in breast cancer cells. To this end, we overexpressed CEMIP in MDA-MB-231 cells and assessed both the wound healing rate and the cell proliferation rate. As anticipated, a high level of CEMIP did not influence cell proliferation, but it did markedly enhance cell migration (Fig. 4, N to P, and fig. S3F). This observation aligns with our previous study, which indicated that CEMIP plays a critical role in BACH1-mediated metastasis across various cancer types (*26*). Together, these data indicate the essential role of BACH1 in regulating pro-metastatic genes during glucose starvation and demonstrate that CEMIP overexpression significantly enhances cell migration, implicating its importance in Keto diet–induced metastasis.

### BACH1 physically interacts with ATF4

**Fig. 5.**
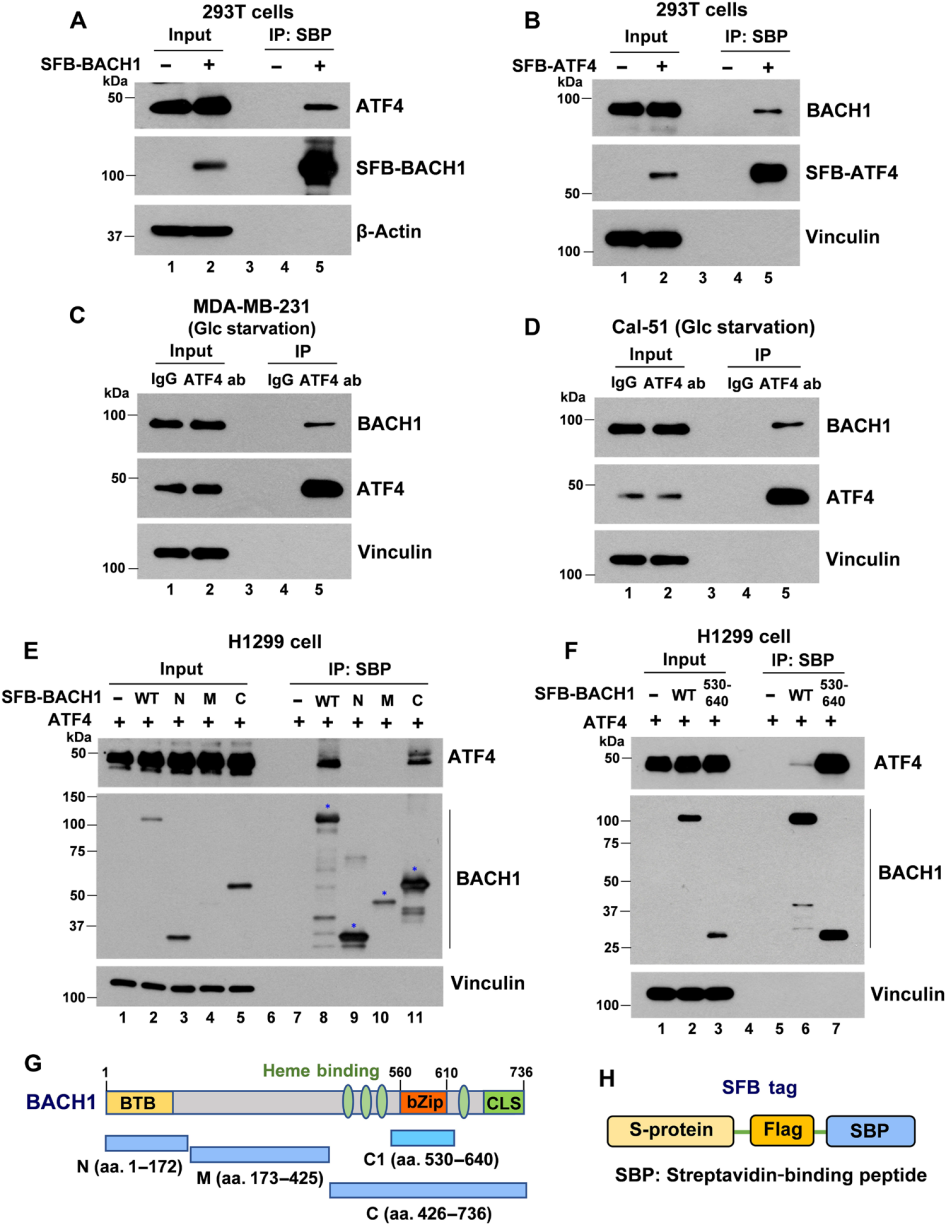
BACH1 physically interacts with ATF4. (**A**) Co-immunoprecipitation (Co-IP) of SFB-tagged BACH1 and endogenous ATF4 in 293T cells. Following transfection with the SFB-BACH1 plasmid, cells were lysed with 1% NP-40 lysis buffer after 24 hours. Subsequent immunoprecipitation was carried out using streptavidin-binding peptide (SBP) beads. SFB, S-protein–Flag–Streptavidin–binding peptide. (**B**) Co-IP of SFB-tagged ATF4 and endogenous BACH1 in 293T cells. (**C** and **D**) Co-IP of endogenous BACH1 and endogenous ATF4 in MDA-MB-231 (C) and Cal-51 cells (D) under glucose starvation condition using either Ctrl IgG or an ATF4-specific antibody (ab). (**E**) Co-IP of ATF4 with SFB-tagged WT BACH1 and its truncated mutants (N, M, and C) in H1299 cells. (**F**) Co-IP of ATF4 with SFB-tagged WT BACH1 and a truncated mutant containing bZip domain [amino acids (aa.) 530 to 640] in H1299 cells. (**G**) Diagram of BACH1 structure and its truncated derivatives. BTB, broad-complex, tramtrack, and bric-a-brac; bZip, basic leucine zipper; CLS, cytoplasmic localization signal. (**H**) Diagram of the SFB tag, which includes tandem S-protein, Flag, and Streptavidin–binding peptide.

To explore the mechanisms by which BACH1-induced pro-metastatic genes are up-regulated under glucose starvation, we hypothesized that a specific protein might be induced during glucose starvation, enhancing BACH1’s transcriptional activity. Activating transcription factor 4 (ATF4) is a stress-responsive transcription factor that plays a pivotal role in cellular responses to various stress conditions, including nutrients deprivation, oxidative stress, and endoplasmic reticulum (ER) stress (*43*). Given that the ATF4 protein level is markedly increased during glucose starvation (as shown in Fig. 4, L and M), we explored a potential relationship with BACH1. Thus, we investigated whether there is a physical interaction between BACH1 and ATF4. To that end, we performed co-immunoprecipitation (Co-IP) of S-protein–Flag–Streptavidin–binding peptide (SFB)-tagged BACH1 with endogenous ATF4 in 293T cells (Fig. 5, A and H). As depicted in Fig. 5A, after transient transfection of the SFB-BACH1 plasmid into cells, ATF4 was clearly detected in the immunoprecipitate of SFB-BACH1. Subsequently, we reversed the Co-IP procedure by transfecting SFB-ATF4 and detecting endogenous BACH1 in the ATF4 complex, which further confirmed their interaction (Fig. 5B).

To explore the natural interaction between BACH1 and ATF4 under glucose-deprived conditions, we carried out endogenous Co-IP in MDA-MB-231 and Cal-51 cells using Ctrl immunoglobulin G (IgG) and ATF4-specific antibodies. As shown in Fig. 5 (C and D), an interaction between ATF4 and BACH1 is evident under physiological conditions.

To characterize further the interaction between BACH1 and ATF4 in human cells, we transfected H1299 cells with an ATF4 expression vector, either in the presence or in the absence of vectors encoding different domains of SFB-BACH1 (N, 1 to 172; M, 173 to 425; and C, 426 to 725) as depicted in Fig. 5G. Co-IP result in Fig. 5E indicates that ATF4 interacts with the C-domain of BACH1. Subsequent analysis further delineated the binding regions, confirming that the bZip domain of BACH1 is the primary region for interaction with ATF4, as presented in Fig. 5F. These results suggest a specific binding between BACH1 and ATF4 and emphasize the bZip domain of BACH1 as the principal interacting domain with ATF4.

### Glucose deprivation–induced up-regulation of pro-metastatic genes and Keto diet–induced lung metastasis depend on both ATF4 and BACH1

**Fig. 6.**
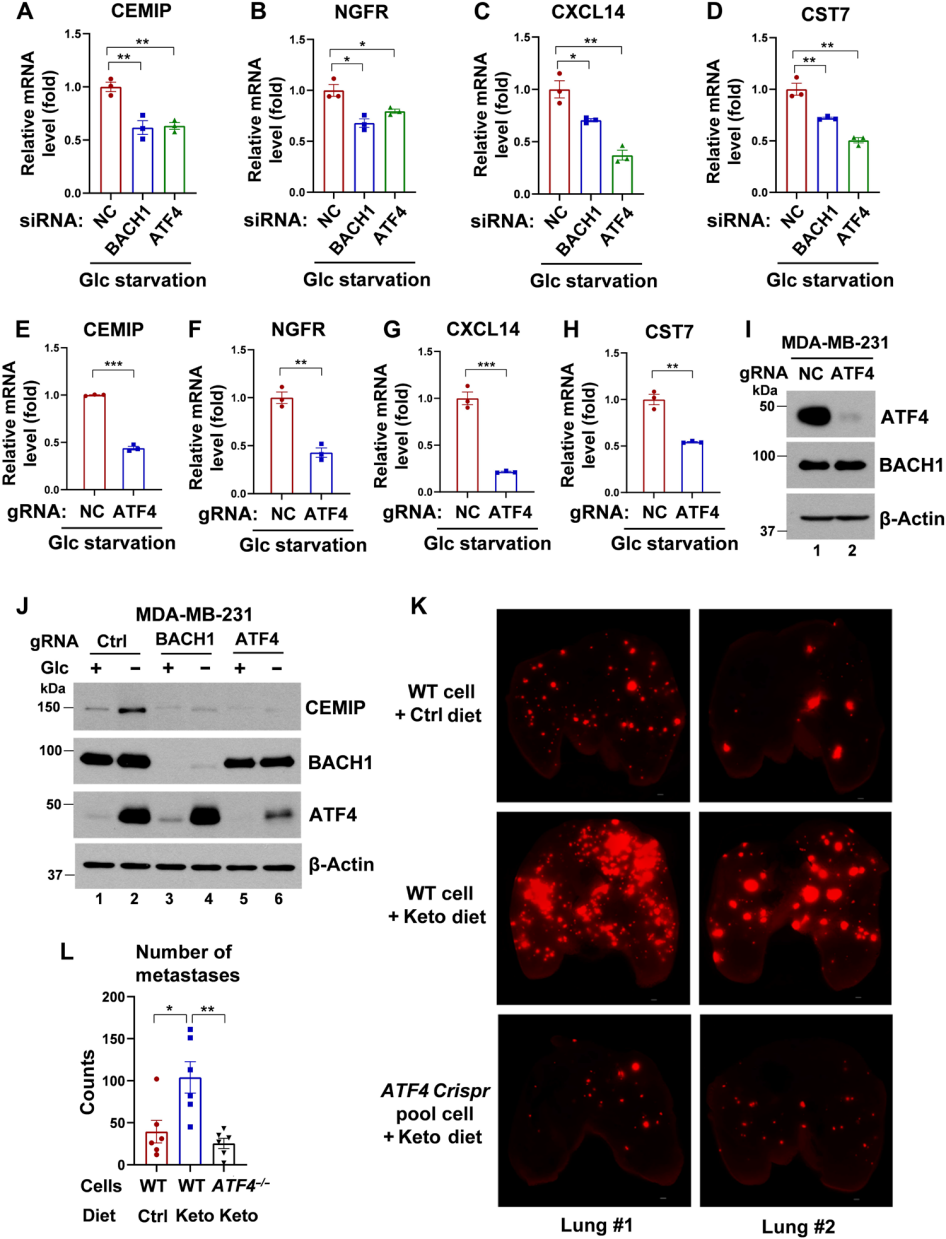
Knockdown or knockout of endogenous ATF4 abrogates BACH1-mediated transcriptional activation and suppresses its effect on tumor metastasis induced by the Keto diet. (**A** to **D**) qPCR analysis of CEMIP (A), NGFR (B), CXCL14 (C), and CST7 (D) expression levels in MDA-MB-231 cells transfected with either negative Ctrl (NC), BACH1, or ATF4 siRNA, under glucose starvation condition (*n* = 3). (**E** to **H**) qPCR analysis of CEMIP (E), NGFR (F), CXCL14 (G), and CST7 (H) expression levels in MDA-MB-231 cells transfected with either negative Ctrl (NC) or ATF4 guide RNA (gRNA), under glucose starvation condition (*n* = 3). (**I**) Western blot analysis of ATF4 expression in MDA-MB-231 cells transfected with either negative Ctrl (NC) or ATF4 gRNA under glucose starvation condition, related to (E) to (H). (**J**) Western blot analysis of CEMIP, BACH1, and ATF4 expression in MDA-MB-231 cells (either Ctrl, BACH1, or ATF4 Crispr pool) incubated with DMEM containing glucose (4.5 or 0 g/liter) for 32 hours. (**K**) Representative images of the lungs infiltrated by MDA-MB-231 WT or ATF4 Crispr pool cells carrying the tomato fluorescence gene. Nude mice were placed on either the Ctrl or Keto diet 2 days before tumor cell injection. One million cells were administered to the nude mice via tail vein injection, and lungs were isolated in the seventh week for imaging. Scale bars, 500 μm. (**L**) Quantitative analysis of the number of metastases in the lungs, related to (K), using BZ-X800 Analyzer software. *n* = 6 mice for each group. In (A) to (H) and (L), data represent means ± SEM; *n* = 3 technical replicates for (A) to (H); *P* values were calculated using unpaired two-tailed Student’s *t* test. *P < 0.05, **P < 0.01, and ***P < 0.001.

Given the physical interaction between BACH1 and ATF4, we next investigated whether the BACH1-dependent expression of pro-metastatic genes under glucose deprivation is also ATF4-dependent. Using qPCR analysis, we found that, under glucose deprivation conditions, the expression levels of CEMIP, CXCL14, CST7, and NGFR in MDA-MB-231 cells diminished when either BACH1 or ATF4 was knocked down by small interfering RNA (siRNA; Fig. 6, A to D, and fig. S4, A and B). This critical involvement of ATF4 was further corroborated by using Crispr-Cas9 method to knock out ATF4. This led to a comparable decline in the expression of the aforementioned genes, emphasizing the important influence of ATF4 on mediating the transcriptional shifts triggered by glucose deprivation (Fig. 6, E to I). Consistent with these findings, Western blot analyses provided additional evidence. An apparent increase in CEMIP expression during glucose deprivation was significantly reduced in ATF4-depleted MDA-MB-231 cells (Fig. 6J).

Transitioning to in vivo studies using the tail vein injection model with MDA-MB-231 cells, we observed that ATF4 protein levels were increased in tumor tissues in Keto diet group (fig. S4C). ATF4-depleted MDA-MB-231 cells formed fewer lung metastasis compared to WT cells in Keto diet groups (Fig. 6, K and L). This highlights the crucial role of ATF4 in Keto diet–induced metastasis of breast cancer cells. Together, our results underscore the pivotal role of ATF4, in collaboration with BACH1, in shaping the transcription of pro-metastatic genes and metastatic capacity of breast cancer cells under Keto diet condition.

### Glucose deprivation–induced ATF4 expression enhances the docking of BACH1 on its pro-metastatic targets

**Fig. 7.**
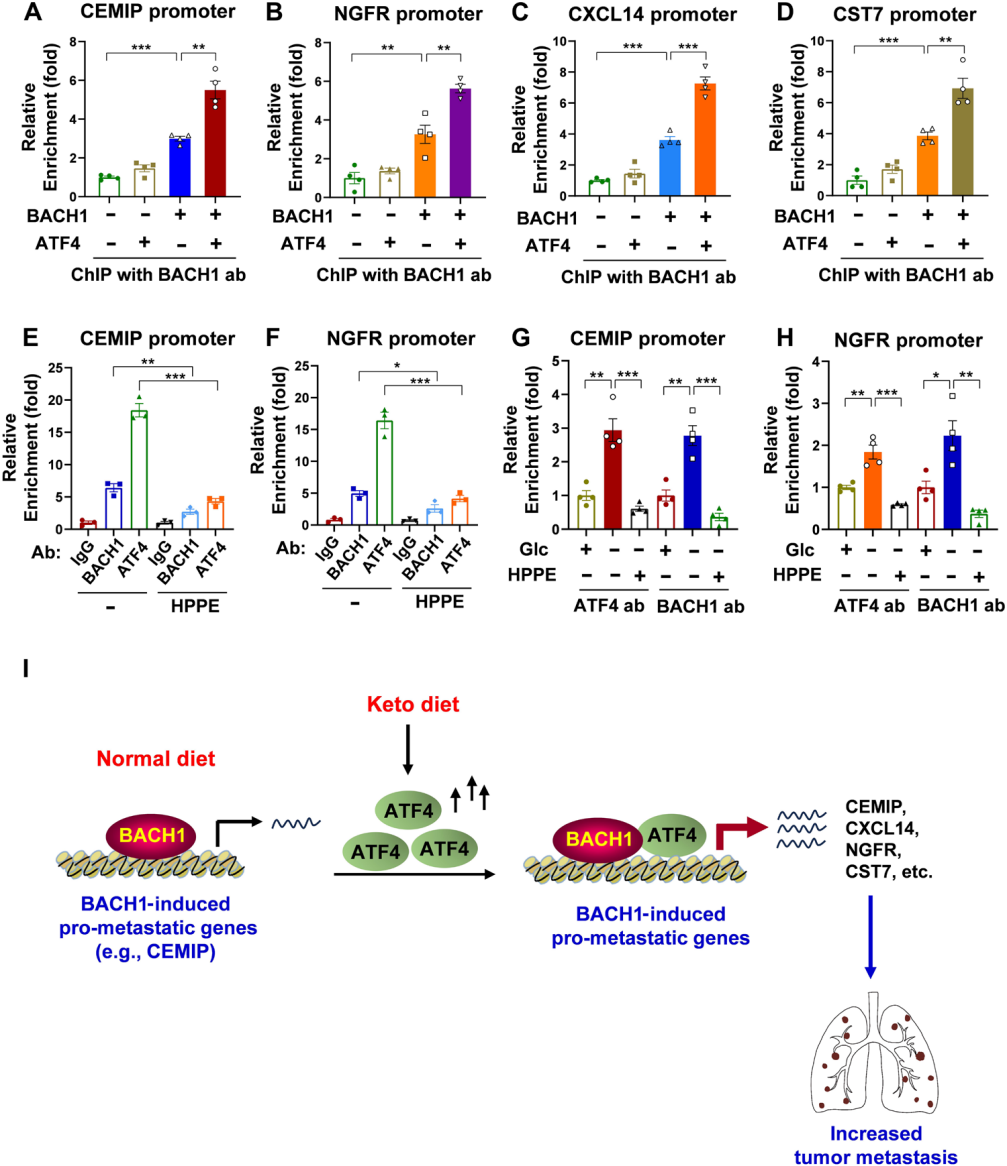
Glucose deprivation–induced ATF4 expression enhances the docking of BACH1 on its pro-metastatic targets. (**A** to **D**) Chromatin immunoprecipitation (ChIP) analysis of BACH1 recruitment to the promoters of CEMIP (A), NGFR (B), CXCL14 (C), and CST7 (D) in MDA-MB-231 *BACH1^−/−^* cells transfected with Ctrl, ATF4, BACH1, or a combination of BACH1 + ATF4 plasmids (*n* = 4). (**E** and **F**) ChIP analysis of the recruitment of BACH1 or ATF4 to the promoters of CEMIP (E) and NGFR (F) in MDA-MB-231 cells treated with/without 2.5 μM HPPE (*n* = 3). (**G** and **H**) ChIP analysis in MDA-MB-231 cells under glucose starvation and non-starvation conditions, examining the recruitment of BACH1 or ATF4 to the promoters of CEMIP (G) and NGFR (H) in the presence or absence of HPPE (*n* = 4). Glc (glucose), 4.5 g/liter; HPPE, 2.5 μM. (**I**) Working model for Keto diet–induced pro-metastatic effect. In normal diet conditions, BACH1 can induce the expression of a number of pro-metastatic genes including *CEMIP*, *CXCL14*, *NGFR*, and *CST7*. However, upon the Keto diet, the ATF4 protein level is markedly induced. Through direct interacting with BACH1, ATF4 is recruited to pro-metastatic target promoters and significantly enhances the DNA binding activity of BACH1 on those promoters, leading to increased expression of these pro-metastatic genes and lung metastasis. When BACH1 is deficient, although ATF4 is induced under Keto diet condition, it is unable to efficiently bind to the promoter of BACH1-regulated pro-metastatic genes, leading to attenuated lung metastasis. In (A) to (H), data represent means ± SEM; *n* = 3 or 4 technical replicates; *P* values were calculated using unpaired two-tailed Student’s *t* test. *P < 0.05, **P < 0.01, and ***P < 0.001.

To clarify the interplay between BACH1 and ATF4 in the regulation of the expression of BACH1-dependent pro-metastatic genes, we conducted a chromatin immunoprecipitation (ChIP) analysis on MDA-MB-231 *BACH1^−/−^* cells that were transiently transfected with either ATF4, BACH1, or both plasmids. As a Ctrl, expressing ATF4 alone in BACH1 knockout cells did not influence BACH1 recruitment. In contrast, BACH1 by itself was effectively recruited to the promoters of CEMIP, NGFR, CXCL14, and CST7 (Fig. 7, A to D). Co-transfection of BACH1 and ATF4 plasmids showed a marked enhancement in the recruitment of BACH1 to the promoters of these genes, suggesting a synergy between ATF4 and BACH1 (Fig. 7, A to D).

In MDA-MB-231 cells under non–glucose starvation conditions, both BACH1 and ATF4 can be recruited to the promoters of CEMIP, NGFR, CXCL14, and CST7 (Fig. 7, E and F, and fig. S5, A and B). However, when the binding of BACH1 to DNA was blocked using HPPE, the recruitment of ATF4 to these genes’ promoters was also attenuated (Fig. 7, E and F, and fig. S5, A and B). This finding suggests that ATF4’s recruitment to these promoters is dependent on BACH1.

ChIP analysis under glucose starvation conditions further revealed interesting dynamics for BACH1 and ATF4 recruitment to the promoters of CEMIP, NGFR, CXCL14, and CST7 (Fig. 7, G and H, and fig. S5, C and D). Under glucose starvation, the recruitment of ATF4 to these gene promoters markedly increased, which was inhibited by the BACH1 inhibitor, HPPE. Concurrently, the recruitment of BACH1 to these gene promoters also had a notable rise, which was similarly blocked by HPPE. These results suggest that ATF4 acts as a coactivator for BACH1 in activation of those pro-metastatic targets. Upon glucose deprivation, the levels of ATF4 are markedly induced; through direct interacting with BACH1, ATF4 is recruited to pro-metastatic target promoters and also significantly enhances the DNA binding activity of BACH1 on those promoters, ultimately leading to augmented transcription of these genes (refer to the working model in Fig. 7I).

### Keto diet serves an important role in the regulation of BACH1/ATF4 function in vivo

**Fig. 8.**
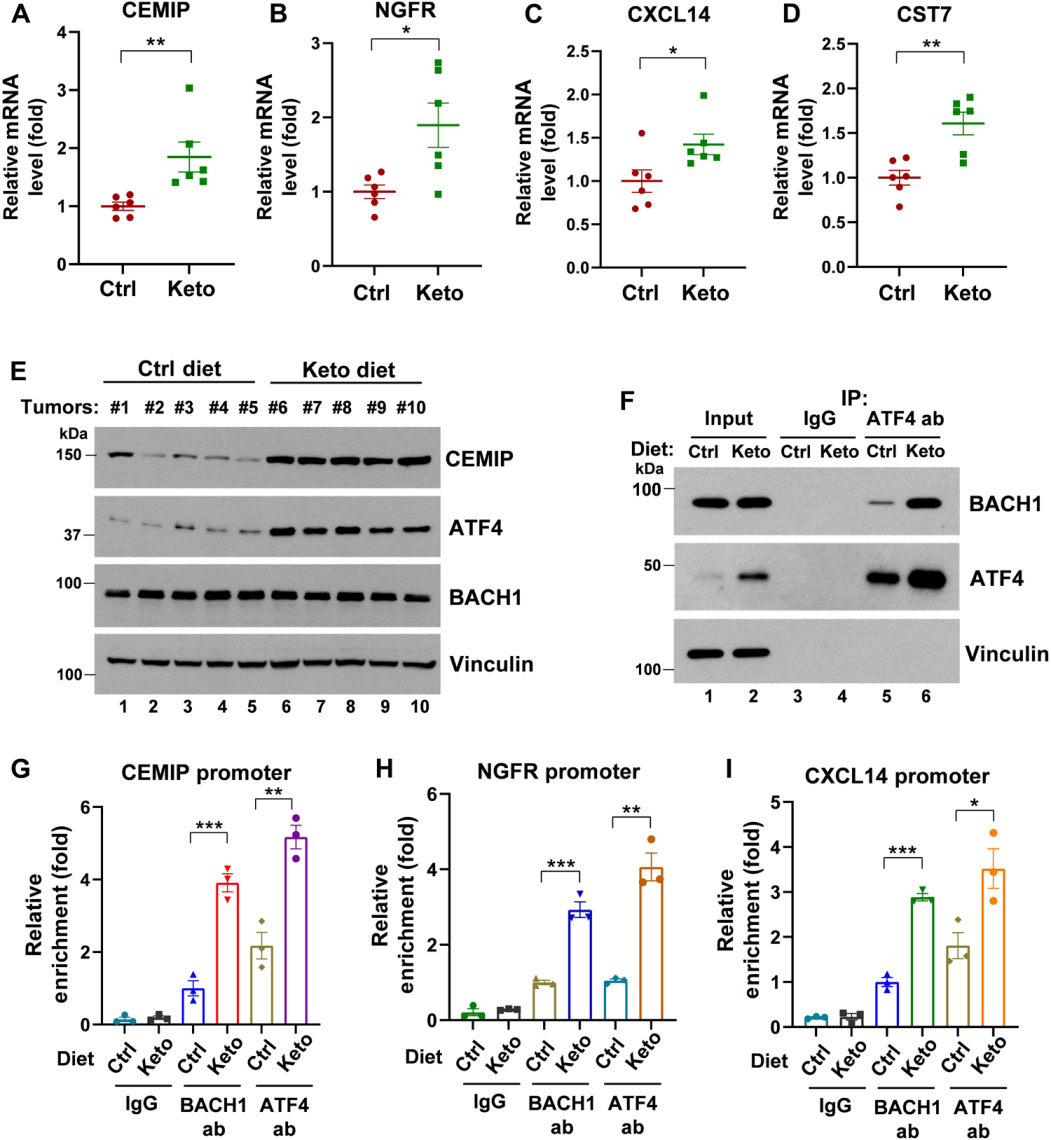
In vivo analysis of Keto diet on BACH1/ATF4 function in an MDA-MB-231 cell xenograft model. Nude mice were inoculated subcutaneously (i.s.) with MDA-MB-231 cells and then placed on Ctrl or Keto diet for 3 weeks. Tumors were collected for qPCR, Western blot, Co-IP, and ChIP analysis. (**A** to **D**) qPCR analysis of BACH1-induced pro-metastatic targets CEMIP (A), NGFR (B), CXCL14 (C), and CST7 (D). *n* = 6 mice. (**E**) Western blot analysis of BACH1 target CEMIP expression in tumor tissues. *n* = 5 tumors for each group. (**F**) Co-IP analysis of BACH1 and ATF4 in tumor tissues. Tumor tissue lysates were IP with IgG or ATF4-specific antibody, followed by Western blot analysis of BACH1 in the IP complex. Three tumors from each group were mix for analysis. (**G** to **I**) ChIP analysis of the recruitment of BACH1 or ATF4 to the promoters of CEMIP (G), NGFR (H), and CXCL14 (I) in tumor tissues under different diets. Fresh tumor tissues from Ctrl and Keto diet groups were minced, homogenized with PBS buffer, and the tumor cells were isolated and subjected to ChIP assay using IgG, BACH1-specific antibody, or ATF4-specific antibody. Three tumors from each group were mix for analysis. In (A) to (D) and (G) to (I), data represent means ± SEM; *n* = 3 technical replicates for (G) to (I); *P* values were calculated using unpaired two-tailed Student’s *t* test. *P < 0.05, **P < 0.01, and ***P < 0.001.

Next, we explored the in vivo effects of the Keto diet on BACH1/ATF4 function in an MDA-MB-231 xenograft tumor model. As shown in Fig. 8 (A to D), upon the Keto diet for 3 weeks, the mRNA levels of BACH1’s pro-metastatic targets, including CEMIP, NGFR, CXCL14, and CST7, were significantly increased in MDA-MB-231 tumors. The Keto diet also increased the protein levels of CEMIP (Fig. 8E). Furthermore, the Keto diet enhanced ATF4 protein levels in tumors and the BACH1-ATF4 interaction in vivo (Fig. 8, E and F). Moreover, ChIP analysis of tumor tissues revealed that the recruitment of BACH1 to the promoters of CEMIP, CXCL14, and NGFR was markedly enhanced in tumors from the Keto diet group compared to the Ctrl group (Fig. 8, G to I). In addition, the recruitment of ATF4 to the promoters of BACH1 targets was also increased in response to the Keto diet (Fig. 8, G to I). These in vivo data further support the working model that we proposed in Fig. 7I.

### The expression of BACH1 and CEMIP has important clinical implications in breast cancer progression

Analysis from the GEPIA2 database shows a marked correlation between mRNA expression of BACH1 and CEMIP and the overall survival rates of patients with invasive breast carcinoma (fig. S6, A and B). Specifically, an elevated expression of BACH1 is inversely associated with the overall survival, suggesting that patients with higher BACH1 expression face decreased survival rates (*P* = 0.024). Likewise, enhanced CEMIP expression is negatively correlated with survival, confirming that higher CEMIP expression results in decreased overall survival (*P* = 0.0018) (fig. S6, A and B). Such observations underscore the potential prognostic value of BACH1 and CEMIP in invasive breast carcinoma.

Using gene chip data from the TNMplot database, a comparison of BACH1 expression levels was conducted across normal breast tissues (*n* = 242), breast tumors (*n* = 7569), and metastatic tumors (*n* = 82) (fig. S6C). The results revealed a marked increase in BACH1 expression in breast tumors and metastatic tumors compared to normal breast tissues. Although not statistically significant (*P* = 0.0578), the median BACH1 expression level in metastatic tumors was notably elevated (597.5) relative to breast tumors (483) (fig. S6C). This pattern hints at a potential link between increased BACH1 expression and the progression of cancer metastasis.

## DISCUSSION

Several preclinical studies have reported that a Keto diet can delay tumor growth in different cancer types, although the emphasized mechanisms vary (*11*, *14*, *44*–*46*). This has been reinforced by a recent clinical trial in which 80 patients with breast cancer undergoing chemotherapy were given either a Keto diet or a Ctrl diet for 12 weeks (*15*). Patients in the Keto diet group with locally advanced types had a better response to chemotherapy, such as reduced tumor size and downstaging, compared to those in the Ctrl diet group. However, there was no evidence to show that Keto diet can prevent or treat metastatic lesions (*15*). On the basis of our findings that the Keto diet may increase the risk of tumor metastasis in breast cancer, it may be more efficient to combine the use of the Keto diet with BACH1 inhibition, as we have proven that BACH1 deficiency can block Keto diet–induced metastasis. BACH1 has been identified as an important pro-metastatic factor in various cancer types, including lung cancer, colorectal cancer, and breast cancer (*17*, *18*, *20*, *22*, *28*). Patient data show that BACH1 expression is significantly higher in breast tumor tissues compared to normal breast tissues, and it is even higher in metastatic tumors of breast cancer (fig. S6C). Therefore, combining a Keto diet with BACH1 inhibition (e.g., using BACH1 inhibitors or proteolysis targeting chimera drugs) might preserve the growth-repressive effect of the Keto diet while eliminating its metastasis-promoting effect.

Because the BACH1-ATF4 complex is specifically required for activation of the prometastatic targets of BACH1, it is possible that the formation of the BACH1-ATF4 heterodimer may play an important role in recognizing specific targets for transcriptional activation. We examined whether the formation of the BACH1-ATF4 heterodimer enables BACH1 to bind the activator protein 1 (AP-1)–related sequence. By using a DNA pull-down assay, we observed that the BACH1-ATF4 heterodimer specifically interacts with the AP-1 DNA fragment but BACH1, MAFG, ATF4 alone, or the BACH1-MAFG heterodimer failed to do so. Although further investigation is warranted for more detailed analysis, these results reveal a previously unknown mechanism by which BACH1 is able to recognize a DNA binding site AP-1 for activating its prometastatic target CEMIP by forming a heterodimer with ATF4 (fig. S7, A and B).

In a recent study, Ferrer *et al.* (*14*) reported that the Keto diet promoted tumor ferroptosis. Moreover, BACH1 has been reported to promote ferroptosis by suppressing ferroptosis-related genes, including *SLC7A11* (*25*, *47*). This raises the possibility that activation of BACH1 by the Keto diet may suppress tumor growth through the down-regulation of SLC7A11 and the activation of ferroptosis. However, we observed that glucose deprivation (mimicking the Keto diet in vitro) up-regulates SLC7A11 expression rather than down-regulating it (Fig. 4L). This result suggests that Keto diet–mediated tumor suppression is unlikely to be due to BACH1-induced ferroptosis. In addition, our recent study has shown that tumor growth and tumor metastasis can be separately regulated. p53-R175H promotes tumor growth by suppressing ferroptosis but facilitates tumor metastasis via up-regulating CEMIP. p53-R175H–mediated regulation of SLC7A11 and ferroptosis does not affect its pro-metastatic function (*26*). Therefore, Keto diet–mediated regulation of ferroptosis and tumor growth does not support the notion that BACH1-mediated ferroptosis is required for its effect in modulating tumor metastasis although further investigations are clearly needed. Instead, our data indicate that pro-metastatic targets induced by BACH1 such as CEMIP play a key role in this process.

Cachexia, a wasting syndrome commonly observed in patients with advanced cancer, is characterized by skeletal muscle wasting and malnutrition (*48*). While it is well-established that cancer metastasis can lead to cachexia, the accelerated cancer spread observed in patients with late-stage cancer with cachexia suggests that cachexia might also exacerbate cancer metastasis (*49*, *50*). The weakened antitumor immunity and heightened systemic inflammation seen in cachexia could partly account for this rapid metastatic spread (*51*, *52*). Given our findings, it is intriguing to hypothesize that low blood glucose level in patients with cachexia might further augment the metastatic potential of the tumors. It is possible that extensive tumor metastases lead to cachexia, which subsequently results in glucose shortage. This state of glucose deprivation might further accelerate tumor metastasis in patients with cachexia. This vicious cycle can eventually render the disease uncontrollable. A deeper understanding of the interplay between cachexia, glucose deprivation, and cancer metastasis could be pivotal for the treatment of patients with advanced-stage cancer in the future.

It is known that many stresses, including nutrient deprivation, ER stress, oxidative stress, ultraviolet (UV) exposure, and hypoxia, can induce ATF4 expression (*43*). In addition, ATF4 is frequently up-regulated in cancer cells (*43*). The most important upstream regulator of ATF4 expression is the eukaryotic initiation factor 2 α subunit (eIF2α). Phosphorylation of eIF2α is a key event in the translation of ATF4 (*53*). eIF2α phosphorylation can be catalyzed by four different stress-induced kinases: protein kinase R (PKR)–like ER kinase (PERK), general Ctrl non-derepressible 2 (GCN2), protein kinase RNA–activated (PKR), and heme-regulated inhibitor (HRI) (*43*). Usually, PERK is activated by ER stress and hypoxia, while GCN2 is activated by nutrient deprivation (e.g., amino acid limitation and glucose starvation) and UV exposure (*54*, *55*). PKR is activated by viral infection, and HRI is activated by heme deprivation and oxidative stress (*56*, *57*). In addition, some stresses can activate ATF4 in a phospho-eIF2α–independent manner. For example, hypoxia can regulate ATF4 stability and transcriptional activity (*58*). Certain drug-induced ER stresses also up-regulate ATF4 transcription (*59*). We have found that a Keto diet induces ATF4 expression in tumor tissues. Given that a Keto diet contains no or very few carbohydrates, it represents a form of nutrient deprivation. Therefore, ATF4 expression induced by Keto diet may be activated by GCN2. Moreover, the Keto diet has been observed to up-regulate ATF4 transcription in normal tissues (*60*, *61*). Thus, the Keto diet may induce ATF4 expression through both transcriptional and translational mechanisms. Further studies are required to investigate the exact mechanisms of ATF4 induction in response to the Keto diet.

In this study, our primary focus is on breast cancer cell lines, exploring the impact of Keto diet on promotion of breast tumor metastasis. We also observed a similar phenomenon in mouse sarcoma cells upon carbohydrate starvation (Fig. 2, D to F). Therefore, we hypothesize that the effect of Keto diet on promoting tumor metastasis might not be limited to breast cancer. Future research is needed to elucidate this potential broader applicability. Moreover, NFE2-like bZIP transcription factor 2 (NFE2L2 or NRF2), a well-known stress response factor, also exhibits increased expression under glucose starvation similar to ATF4, and is known to interact with ATF4 (*62*, *63*). Considering that NRF2 and BACH1 share many target genes (*64*), this suggests the possibility that CEMIP is also a target of NRF2. Therefore, we examined the effect of NRF2 on CEMIP expression. As expected, knockdown of BACH1 in either Cal-33 cells or BT-549 cells down-regulated CEMIP expression; unexpectedly, knockdown of NRF2 in those cells up-regulated CEMIP expression (fig. S8, A and B). Although further investigations are clearly required, these results indicate that, similar to the effects on SLC7A11 by NRF2 versus BACH1, NRF2 apparently plays a different role in modulating CEMIP expression. Investigating the role of NRF2 in Keto diet–induced metastasis will be an interesting direction for future research.

## MATERIALS AND METHODS

The study research complies with all relevant ethical regulations. All experimental protocols were approved by the Institutional Animal Care and Use Committee of Columbia University.

### Reagents

BACH1 inhibitor HPPE (*42*) was synthesized by Enamine. Hemin was from Sigma-Aldrich (catalog no. 51280). Ctrl diet (TD.150345) and Keto diet (TD.160153) were from Teklad Diets, Inotiv (see table S1 for details). Glucose solution was from Thermal Scientific (catalog no. A2494001), glucose-free Dulbecco’s modified Eagle’s medium (DMEM) was from Thermal Scientific (catalog no. 11966025), 3-hydroxybutyric acid was from Sigma-Aldrich (catalog no. 166898), and decanoic acid was from Sigma-Aldrich (catalog no. PHR3529). Other key reagents are listed in tables S2 and S3 or mentioned below.

### Cell lines

293T (human, no. CRL-3216), H1299 (human, no. CRL-5803), T-47 (human, no. HTB-133), and 4T1 (mouse, no. CRL-2539) cells were from American Type Culture Collection; Cal-51 (human, no. ACC 302) and Cal-148 (human, no. ACC 460) were from DSMZ. Mouse sarcoma cells were derived from spontaneously produced sarcoma tumor from p53^R172H/R172H^ transgenic mice (C57BL/6J, female). MDA-MB-231 cells and mouse sarcoma cells carrying dTomato fluorescence gene were made in our laboratory. All cell lines have been proven to be negative for mycoplasma contamination. No cell lines used in this work were listed in the International Cell Line Authentication Committee (ICLAC) database. All cells were cultured in a 37°C incubator with 5% CO_2_. All cancer cells, except for 4T1 cells, are cultured in high-glucose DMEM (Corning, catalog no. 10-013-CV) supplemented with 10% fetal bovine serum and 1% penicillin-streptomycin (Thermo Fisher Scientific, catalog no. 15140122), unless otherwise specified. 4T1 cells are cultured in RPMI 1640 medium (Gibco, catalog no. 11875-093).

### Tumor growth and metastasis models

BALB/c NU/NU nude mice (CAnN.Cg-*Foxn1nu/*Crl, female, 6 weeks) and BALB/c mice (BALB/cAnNCrl inbred, female, 6 weeks) were purchased from Charles River Laboratories. All studies were compliant with all relevant ethical regulations for animal experiments. All of the experimental protocols were approved by the Institutional Animal Care and Use Committee of Columbia University.

#### For xenograft model

MDA-MB-231 cells (1.0 × 10^6^) were mixed with Matrigel (Corning, catalog no. 354248) at 1/1 ratio (v/v) and injected subcutaneously into BALB/c nude mice (CAnN.Cg-*Foxn1nu/*Crl, 6 weeks, female). After tumor inoculation, mice were fed with Ctrl diet or Keto diet for 6 weeks, and, then, mice were euthanized, and the tumors were weighed and imaged. Fresh tumor samples were subjected to qPCR analysis, Western blot analysis, Co-IP, and ChIP analysis.

#### For tail vein injection lung metastasis model

BALB/c nude mice (CAnN.Cg-*Foxn1nu/*Crl, female, 6 weeks) were pre-fed with Ctrl or Keto diet for 2 days, followed by tail vein injection of 1 million to 1.5 million MDA-MB-231 cells or mouse sarcoma cells (*p53^R172H/R172H^*) carrying dTomato fluorescence gene. Six to 7 weeks later, mice were euthanized, and lungs were isolated for imaging using KEYENCE BZ-X800 fluorescence microscope. Then, lungs were fixed by 10% formalin and subjected to H&E staining and immunohistochemical staining. As to 4T1 cells, BALB/c mice (BALB/cAnNCrl inbred, female, 6 weeks) were pre-fed with Ctrl or Keto diet for 2 days, followed by tail vein injection of 0.1 million to 0.2 million 4T1 cells carrying luciferase gene. Two to 3 weeks later, mice were intraperitoneally injected with luciferase substrate d-luciferin (PerkinElmer, catalog no. 122799) and imaged on IVIS Spectrum Optical Imaging System.

#### For mammary fat pad injection lung metastasis model

BALB/c mice (BALB/cAnNCrl inbred, female, 6 weeks) were inoculated with 0.5 million to 1 million luciferase-expressing 4T1 cells via the mammary fat pad at gland #4. After tumor cell inoculation, mice were fed with either the Ctrl or Keto diet. Three to 4 weeks later, mice were imaged using the IVIS Spectrum Optical Imaging System. At the end of experiment, mice were euthanized, and lungs were isolated for H&E staining, and primary tumors were isolated for weighing.

### Western blotting and immunoprecipitation

Cells were lysed in RIPA buffer [10 mM tris Cl (pH 8.0), 150 mM NaCl, 1% Triton X-100, 1% Na-deoxycholate, 1 mM EDTA, 0.05% SDS, and fresh 1× proteinase inhibitor], and protein concentrations were quantified using the Protein Assay Dye Reagent (Bio-Rad, catalog no. 5000006). Samples, once normalized for protein content, were subjected to SDS-PAGE, and proteins were subsequently electro-transferred to 0.45-μm nitrocellulose membranes (Thermo Fisher Scientific, catalog no. 88018). Following transfer, membranes were incubated with primary antibodies either overnight at 4°C or for 1.5 hours at room temperature and then incubated with horseradish peroxidase (HRP)–conjugated secondary antibodies at room temperature for 1 to 1.5 hours and visualized on autoradiographic films after incubation with either Pierce ECL Western Blotting Substrate (Thermo Fisher Scientific, catalog no. 32106) or SuperSignal West Dura Extended Duration Substrate (Thermo Fisher Scientific, catalog no. 34076).

For endogenous IP, cells were lysed in 1% NP-40 lysis buffer containing 1% NP-40, 150 mM NaCl, 50 mM tris-HCl, and 1 mM EDTA. Lysates were incubated overnight with 2 to 4 μg of either IgG or the specific primary antibody, followed by a 3- to 4-hour incubation with protein A agarose (GE Healthcare, catalog no. 17-0780-01) that had been pre-blocked with 0.1% bovine serum albumin. Subsequently, beads were washed using the 1% NP-40 lysis buffer and bound proteins were eluted by incubating with 0.1 M glycine (pH 2.6) for 15 min at room temperature. For streptavidin-binding peptide IP, cells were similarly lysed in 1% NP-40 lysis buffer, and the lysates were incubated overnight with streptavidin beads (Thermo Fisher Scientific, catalog no. 45-000-279). Following this, the beads were washed using the 1% NP-40 lysis buffer, and proteins were eluted by incubating with biotin (2 mg/ml) for 1 hour at room temperature. Antibodies used in this study are listed in table S2.

### Quantitative PCR

Total RNA was isolated using TRIzol (Thermo Fisher Scientific, catalog no. 15596018) following the manufacturer’s instructions. Reverse transcription was performed to generate cDNA using the SuperScript IV VILO Master Mix (Thermo Fisher Scientific, catalog no. 11756500). Quantitative PCR assays were conducted on a 7500 Fast Real-Time PCR System (Applied Biosystems) following the standard protocol. qPCR primers used in this study are listed in table S3.

### RNA interference and Crispr genome editing

Cells were plated at 30 to 40% density 1 day before siRNA transfection and then transfected with 80 nM siRNA pool using Lipofectamine 3000 (Thermo Fisher Scientific, catalog no. L3000008). Cells were collected at 36 to 48 hours after transfection and subjected to functional assays. siRNAs used in this study are listed in table S3.

As to Crispr editing, TrueGuide Synthetic guide RNAs (gRNAs; Thermo Fisher Scientific, catalog nos. A35533 and A35534) were co-transfected with TrueCut Cas9 Protein v2 (Thermo Fisher Scientific, catalog no. A36498) using Lipofectamine CRISPRMAX (Thermo Fisher Scientific, catalog no. CMAX00008) according to the manufacturer’s protocol. Forty-eight hours later, Crispr efficiency was determined by Western blot analysis. The Crispr pool cells were used for subsequent functional assays or selected for monoclonal isolation. Specifically, the Crispr pool cells were seeded in 10-cm dishes at a density of 100 to 200 cells per dish to generate monoclones. After 1 to 2 weeks, monoclonal colonies were isolated and transferred to 12-well plates. Subsequent identification was performed by Western blot analysis. gRNAs used in this study are listed in table S3.

### ChIP assay

Cells were cross-linked with 1% formaldehyde for 10 min at room temperature, and the reaction was neutralized by adding glycine to a final concentration of 0.125 M. After washing twice with cold phosphate-buffered saline (PBS), cells were harvested and resuspended in ChIP lysis buffer A [10 mM tris-Cl (pH 8.0), 85 mM KCl, 0.5% NP-40, 5 mM EDTA, and 1× proteinase inhibitor]. Following a 10-min incubation on ice, nuclei were harvested and resuspended in ChIP lysis buffer B [1% Triton X-100, 10 mM tris-HCl (pH 8.0), 150 mM NaCl, 5 mM EDTA, 0.1% SDS, 0.1% sodium deoxycholate, and 1× protease inhibitor] and incubated for 10 min at 4°C. Lysates were sonicated for 4 min to fragment chromatin and then centrifuged. Supernatants were collected, aliquoted equally, and incubated with the indicated antibodies overnight at 4°C. Protein A–coated magnetic beads (Diagenode, catalog no. C03010020) were then added to each sample and incubated for 4 to 5 hours at 4°C. Beads were sequentially washed with TSE I [20 mM tris-HCl (pH 8.0), 2 mM EDTA, 150 mM NaCl, 0.1% SDS, and 1% Triton X-100], TSE II [20 mM tris-HCl (pH 8.0), 2 mM EDTA, 500 mM NaCl, 0.1% SDS, and 1% Triton X-100], buffer III [10 mM tris-HCl (pH 8.0), 1 mM EDTA, 0.25 M LiCl, 1% DOC, and 1% NP-40], and buffer TE [10 mM tris-HCl (pH 8.0) and 1 mM EDTA]. Binding components were eluted in 1% SDS and 0.1 M NaHCO_3_, and reverse cross-linking was carried out at 65°C overnight. DNA was purified using the PCR Purification Kit (QIAGEN, 28106), and qPCR was conducted to determine the relative enrichment of each protein at the indicated gene promoters. For the tumor tissue ChIP assay, fresh tumor tissues from different groups were minced into small pieces and gently homogenized with PBS buffer 10 to 20 times. The mixture was allowed to stand for 1 to 2 min to let undissociated tissue fragments settle. Then, the tumor cell suspension was collected for the ChIP assay, using conditions similar to those applied to cell lines. ChIP qPCR primers used in this study are listed in table S3.

### RNA-seq and bioinformatics

MDA-MB-231 WT cells (two individual clones) and *BACH1^−/−^* cells (three individual clones) were incubated with high-glucose (4.5 g/liter) DMEM or glucose-free (0 g/liter) DMEM for 40 hours. Total RNA was prepared using TRIzol (Thermo Fisher Scientific, catalog no. 15596018). The RNA quality was evaluated by Bioanalyzer (Agilent) and confirmed that the RNA integrity number (RIN) > 8. Before performing RNA-seq analysis, a small aliquot of each sample was analyzed by qPCR to confirm that positive Ctrl genes were expressed normally. RNA-seq analysis was performed at the Columbia Genome Center. Specifically, from total RNA samples, mRNAs were enriched by poly-A pull-down and then processed for library preparation by using the Illumina TruSeq chemistry. Libraries were then sequenced using the Illumina NovaSeq 6000. Samples were multiplexed in each lane and yielded targeted number of single-end 100–base pair reads for each sample. Real time analysis (RTA) (Illumina) was used for base calling and bcl2fastq (version 2.19) was used for converting BCL to fastq format, coupled with adaptor trimming. Pseudoalignment was performed to a kallisto index created from transcriptome Human:GRCh38.p12 using kallisto (0.44.0). The differentially expressed genes under various conditions were analyzed by DESeq2 R package. Heatmap was generated by Heatmapper (www.heatmapper.ca/) by using transcripts per million (TPM) values. The genes listed in the heatmap were selected from the differentially expressed genes based on two criteria: (i) First, by comparing the results from WT and BACH1 knockout cells, we identified genes whose expression was markedly down-regulated. These genes are potential BACH1–up-regulated targets. (ii) We then reviewed the literature to determine which genes are associated with promoting metastasis. If more than one reference reported that a gene is involved in certain steps of metastasis, then we classified it as a pro-metastasis gene.

### Detection of metabolic indicators

Mouse blood glucose levels were determined by OneTouch Verio Reflect meter (LifeScan). Blood β-hydroxybutyrate levels of were detected by KetoSens blood ketone meter (i-SENS USA Inc.). For mouse serum insulin detection, blood was collected from the orbital sinus, allowed to clot, and centrifuged for 20 min at 4°C to obtain serum. Serum insulin was tested using a Ultrasensitive Mouse Insulin enzyme-linked immunosorbent assay kit (SenCrystal Chem, catalog no. 90080).

### H&E and IHC staining

Tumor or tissue samples were fixed in 10% formalin for 24 hours and then transferred to 70% ethanol and subjected to standard dehydration processing for preparing the paraffin wax blocks. Paraffin blocks were sectioned at 4-μm thickness for H&E staining and immunohistochemistry (IHC) staining. Major conditions for ATF4 IHC: Antigens were retrieved at Tris-EDTA buffer (pH 9.0); primary antibody, 1:50 (Cell Signaling Technology, catalog no. 11815) for 2 hours.

### Lentivirus-based gene transfer

293T cells were transiently co-transfected with lentiviral backbone constructs (pUltra-Chili dTomato) and three packaging plasmids pLP1, pLP2, and pLP/VSVG using the Lipofectamine 3000 transfection reagent (Thermo Fisher Scientific, catalog no. L3000008). Lentivirus-containing medium was collected at 48 hours after transfection and supplemented with polybrene (8 μg/ml; Sigma-Aldrich, catalog no. TR-1003). MDA-MB-231 cells and mouse sarcoma cells were infected by replacing the cell culture medium with lentivirus-containing medium and centrifuged at 1800 rpm for 1 hours followed by 37°C culture for 24 hours. Stable cells were sorted by fluorescence-activated cell sorting (FACS) using tomato fluorescence.

### Wound healing assay

For the wound healing assay, cells were seeded in a six-well plate and transfected with Ctrl or CEMIP plasmid. Upon reaching confluence, a straight scratch was carefully introduced using a 200-μl pipette tip. Following the scratch, cells were gently washed with PBS to remove debris and then incubated in fresh culture medium. Initial images of the wound were taken immediately after scratch using an inverted microscope to establish the 0-hour time point, and additional images of the wound area were captured at specified time points (16, 36, and 60 hours). Wound healing areas were quantified using ImageJ software. The wound healing rate was represented as (the wound closure area/the wound area at the initial time point) × 100.

### Statistics

Data were presented as means ± SEM, calculated by GraphPad Prism 9.0 software. Unless otherwise stated, data were analyzed by unpaired two-tailed *t* tests. *P* values of survival curves were calculated by log-rank (Mantel-Cox) test. Differences were considered statistically significant if *P < 0.05, **P < 0.01, or ***P < 0.001. All experiments were repeated at least two or three times. No statistical method was used to predetermine sample size, but our sample sizes are similar to those reported in previous publications (*18*, *26*). No data were excluded from the analyses; mice for xenograft and metastasis experiments were allocated randomly into each experimental group. For in vivo experiments, we used at least five mice per group, which is sufficient to detect meaningful biological difference. For in vitro experiments, unless otherwise stated, *n* = 3 or 4 was chosen as the number of sample size. For both cell-based in vitro experiments and animal studies, the investigators were not blinded during data acquisition and analysis nor to the allocation of experimental groups.
