# Supplementary material for: An unexpected role for the ketogenic diet in triggering tumor metastasis by modulating BACH1-mediated transcription
Source: Sci Adv. 2024 Jun 5;10(23):eadm9481. doi: 10.1126/sciadv.adm9481 (PMC11152127; doi:10.1126/sciadv.adm9481)
Supplement: Supplementary file 1 — Figs. S1 to S8 Tables S1 to S3 [file sciadv.adm9481_sm.pdf]

Science Advances
AAAS

Supplementary Materials for

An unexpected role for the ketogenic diet in triggering tumor metastasis by modulating BACH1-mediated transcription

Zhenyi Su *et al.*

Corresponding author: Wei Gu, wg8@cumc.columbia.edu

Sci. Adv. **10**, eadm9481 (2024)
DOI: 10.1126/sciadv.adm9481

This PDF file includes:

Figs. S1 to S8
Tables S1 to S3
Uncropped Western blots

Figure S1

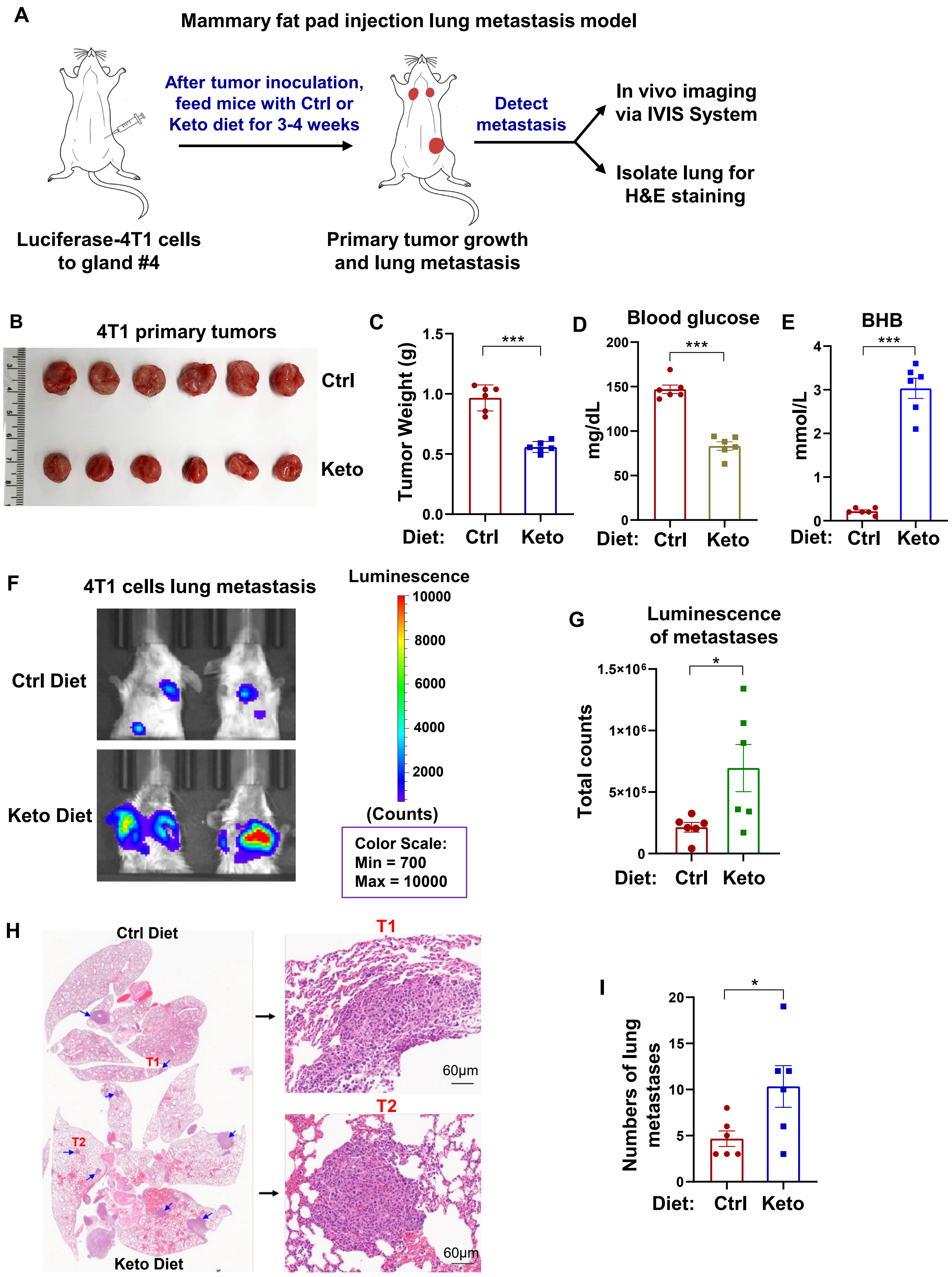
A
Mammary fat pad injection lung metastasis model
After tumor inoculation, feed mice with Ctrl or Keto diet for 3-4 weeks
Detect metastasis
In vivo imaging via IVIS System
Isolate lung for H&E staining
Luciferase-4T1 cells to gland #4
Primary tumor growth and lung metastasis
B
4T1 primary tumors
Ctrl
Keto
C
Tumor Weight (g)
***
Diet: Ctrl Keto
D
Blood glucose
mg/dL
***
Diet: Ctrl Keto
E
BHB
mmol/L
***
Diet: Ctrl Keto
F
4T1 cells lung metastasis
Ctrl Diet
Keto Diet
Luminescence
10000
8000
6000
4000
2000
(Counts)
Color Scale:
Min = 700
Max = 10000
G
Luminescence of metastases
Total counts
*
Diet: Ctrl Keto
H
Ctrl Diet
Keto Diet
T1
T2
60μm
I
Numbers of lung metastases
*
Diet: Ctrl Keto

Figure S1. Keto diet suppresses primary tumor growth but enhances tumor metastasis in 4T1 cells mammary fat pad model, related to Figure 1

(A) Diagram of 4T1 cells mammary fat pad injection lung metastasis model and mouse feeding regimen. BALB/c mice were inoculated with 0.5-1 million luciferase-expressing 4T1 cells via the mammary fat pad at gland #4, then mice were placed on either the Ctrl or Keto diet. Three to four weeks later, mice were imaged using the IVIS Spectrum Optical Imaging System. Lungs were collected for H&E staining at the end of experiment.

(B) 4T1 primary tumors isolated from the mammary fat pads of BALB/c mice 4 weeks post-inoculation and dietary treatment.

(C) Tumor weight of 4T1 primary tumors, related to (B). n=6 mice.

(D) Blood glucose levels after placing mice on Ctrl or Keto diet for 2 weeks. n=6 mice.

(E) Blood β-hydroxybutyrate (BHB) levels after placing mice on Ctrl or Keto diet for 2 weeks. n=6 mice.

(F) In vivo luminescence imaging of lung metastases in BALB/c mice inoculated with luciferase-expressing 4T1 cells via mammary fat pad.

(G) Quantitative analysis of total counts of luminescence in lungs, related to panel (F). n=6 mice for each group.

(H) Representative images of H&E staining of isolated lungs from BALB/c mice inoculated with 4T1 cells via mammary fat pad. Blue arrows indicate metastatic nodules in lungs. T1 and T2 are representative images of metastases in Ctrl and Keto diet groups.

(I) Quantification of lung metastasis counts based on H&E staining. n=6 mice.

In (C-E), (G) and (I), data represent mean ± SEM; p values were calculated using unpaired two-tailed Student's t test. $^{*}p < 0.05$, $^{***}p < 0.001$

Figure S2

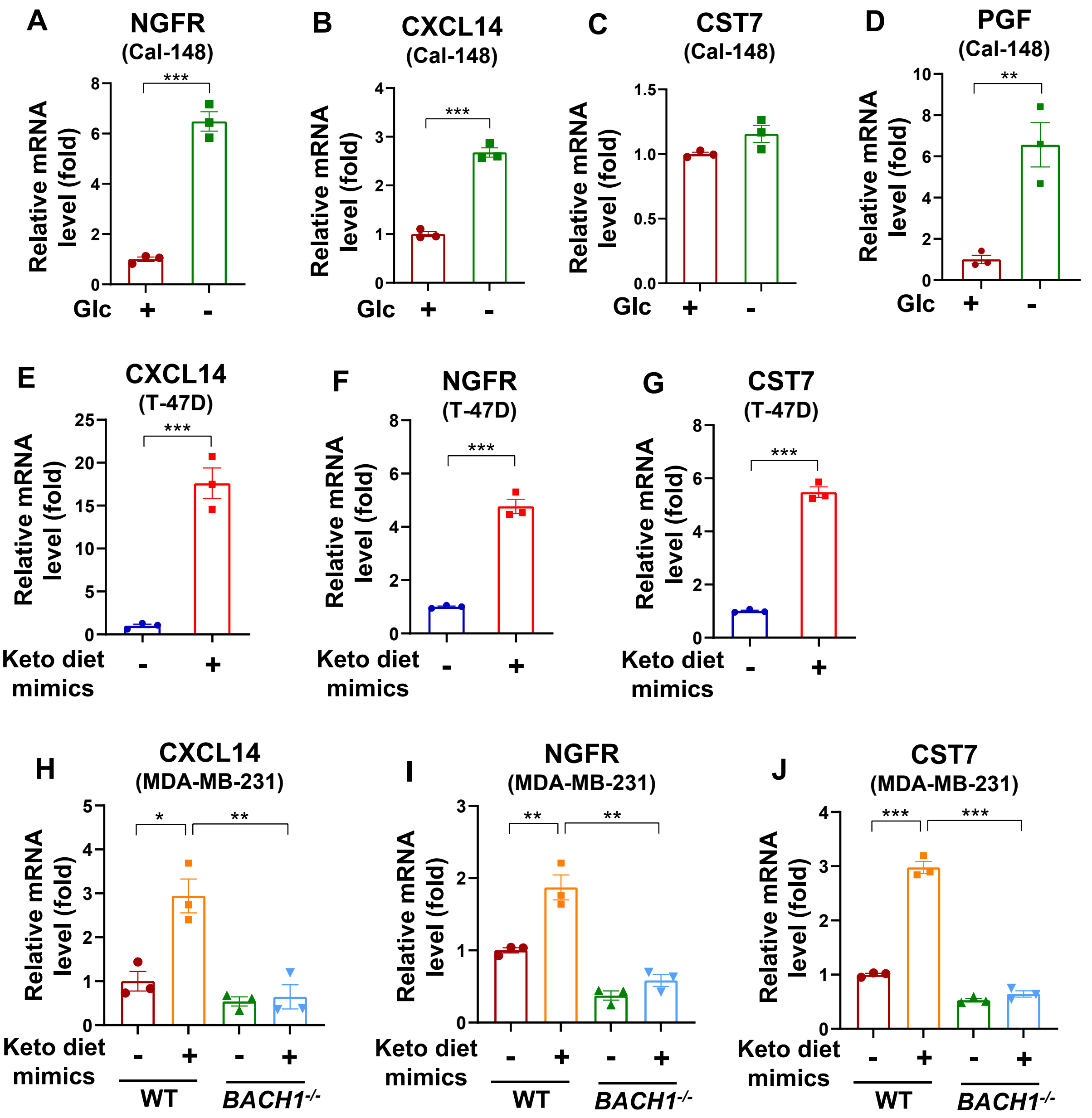

Figure S2. A cluster of BACH1-upregulated pro-metastatic genes are induced upon glucose starvation and Keto diet mimics in vitro, related to Figure 3

(A-D) qPCR analysis of the mRNA expression level of NGFR (A), CXCL14 (B), CST7 (C), and PGF (D) in Cal-148 cells incubated with 4.5g/L or 0g/L glucose for 72h (n=3). Glc, glucose.

(E-G) qPCR analysis of CXCL14 (E), NGFR (F), and CST7 (G) mRNA expression level in T47D cells incubated with or without Keto diet mimics in vitro (0.5g/L glucose + 5mM β-hydroxybutyrate + 100μM decanoic acid) for 4 days. n=3.

(**H-J**) qPCR analysis of CXCL14 (H), NGFR (I), and CST7 (J) mRNA expression level in MDA-MB-231 WT or *BACH1$^{-/-}$* cells incubated with or without Keto diet mimics in vitro (0.5g/L glucose + 5mM β-hydroxybutyrate + 100μM decanoic acid) in FBS-reduced DMEM medium for 4 days. n=3.

In (A-J), data represent mean ± SEM; n=3 technical replicates; p values were calculated using unpaired two-tailed Student's t test. *p < 0.05, **p < 0.01, ***p < 0.001

Figure S3

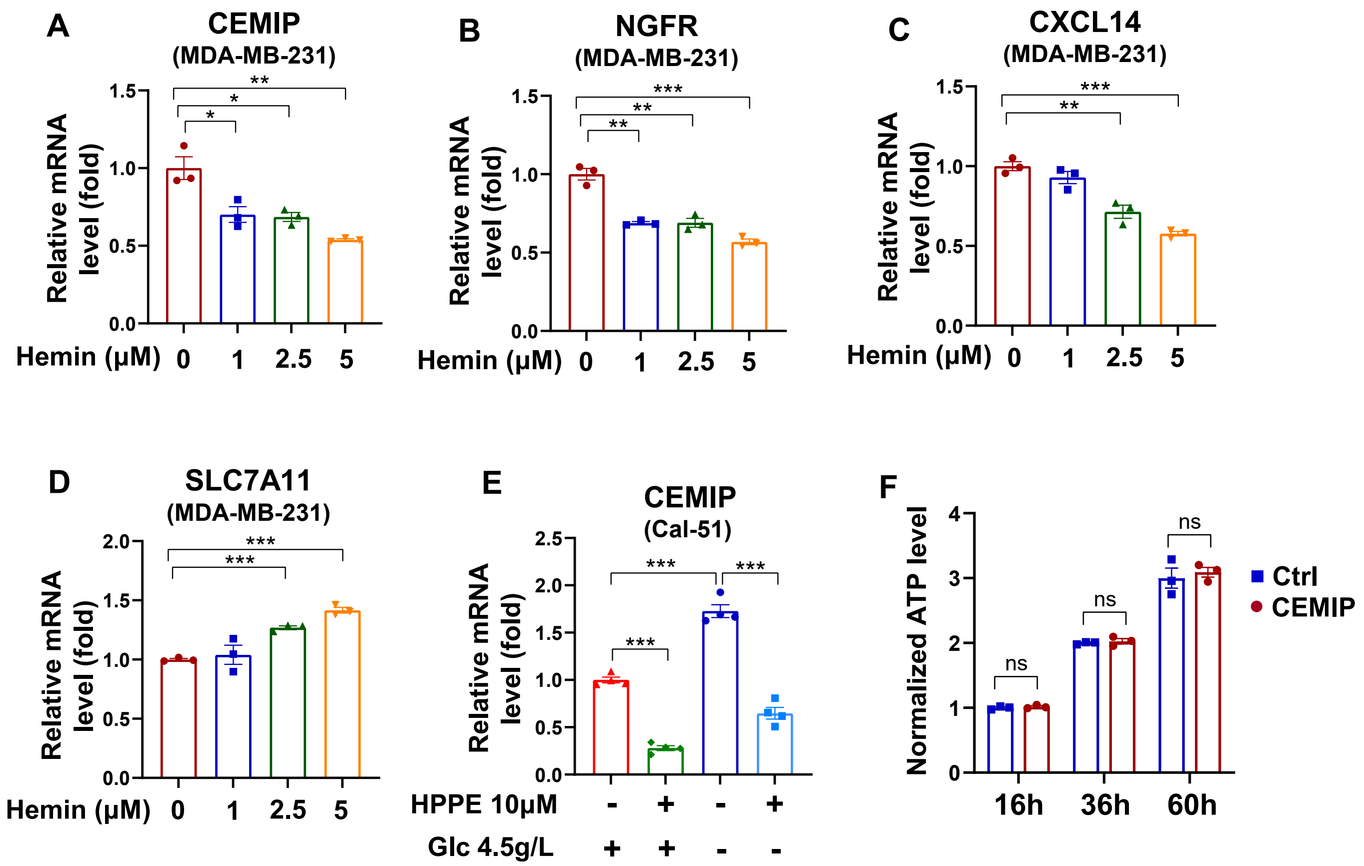

Figure S3. The effect of BACH1 inhibitor, Hemin and HPPE, on the expression of a number of pro-metastatic genes induced by BACH1, related to Figure 4

(A-D) qPCR analysis of mRNA expression levels of CEMIP (A), NGFR (B), CXCL14 (C), and SLC7A11(D) in MDA-MB-231 cells treated with hemin (0, 1, 2.5, and 5μM) for 48h.

(E) qPCR analysis of CEMIP expression levels in Cal-51 cells under normal glucose conditions (4.5g/L) or glucose-free conditions, with or without 10μM HPPE (n=4).

(F) Cell proliferation assay conducted on MDA-MB-231 cells expressing either Ctrl or CEMIP plasmid across various time points (16-60h). ATP levels were detected using the CellTiter-Glo Cell Viability Assay reagent (Promega) and normalized to the control at 16 h (n=3).

In (A-F), data represent mean ± SEM; n=3 or 4 technical replicates; p values were calculated using unpaired two-tailed Student's t test. ns, not significant, $*p < 0.05$, $**p < 0.01$, $***p < 0.001$

Figure S4

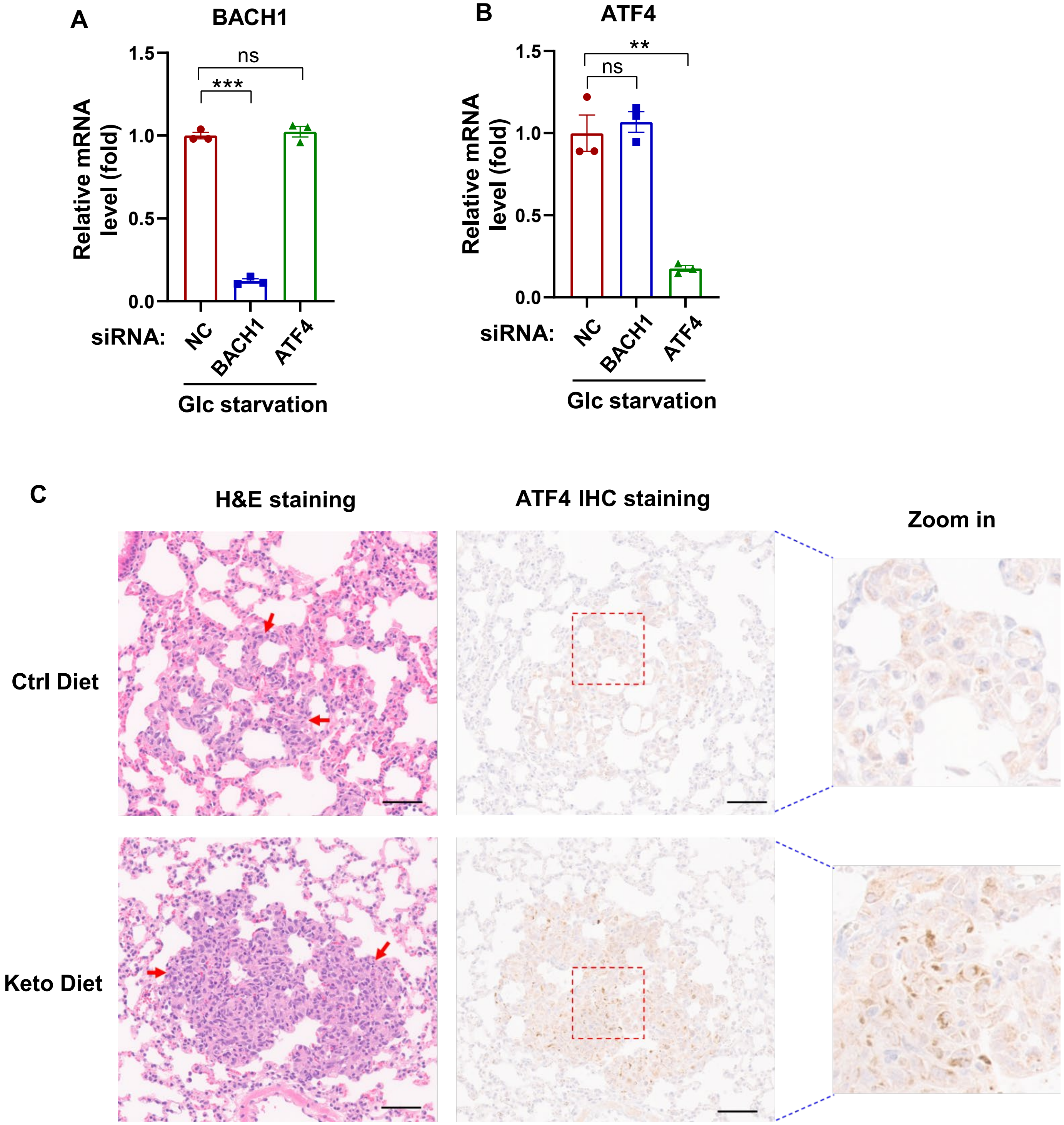

A
BACH1
Relative mRNA level (fold)
1.5
1.0
0.5
0.0
ns
***
siRNA:
NC
BACH1
ATF4
Glc starvation
B
ATF4
Relative mRNA level (fold)
1.5
1.0
0.5
0.0
**
ns
siRNA:
NC
BACH1
ATF4
Glc starvation
C
H&E staining
ATF4 IHC staining
Zoom in
Ctrl Diet
Keto Diet

Figure S4. Knockdown or knockout of endogenous ATF4 abrogates BACH1-mediated transcriptional activation and suppresses its effect on tumor metastasis induced by Keto diet, related to Figure 6.

(A-B) qPCR analysis of the knockdown efficiency in MDA-MB-231 cells transfected with negative control (NC), BACH1, or ATF4 siRNA under glucose starvation conditions, related to Figure 6A-6D (n=3).

(C) H&E staining and immunohistochemistry (IHC) staining of ATF4 in lungs infiltrated with MDA-MB-231 tumors, related to Figure 6K. Nude mice were placed on either the Ctrl or Keto diet 2 days prior to tumor cell injection. One million cells were administered to the nude mice via tail vein, and lungs were isolated in the 7th week for imaging and staining. Red arrow indicates metastatic nodules in lungs. Scale bar, 60μm.

In (A) and (B), data represent mean ± SEM; n=3 technical replicates; p values were calculated using unpaired two-tailed Student's t test. ns, not significant, $**p < 0.01$, $***p < 0.001$

Figure S5

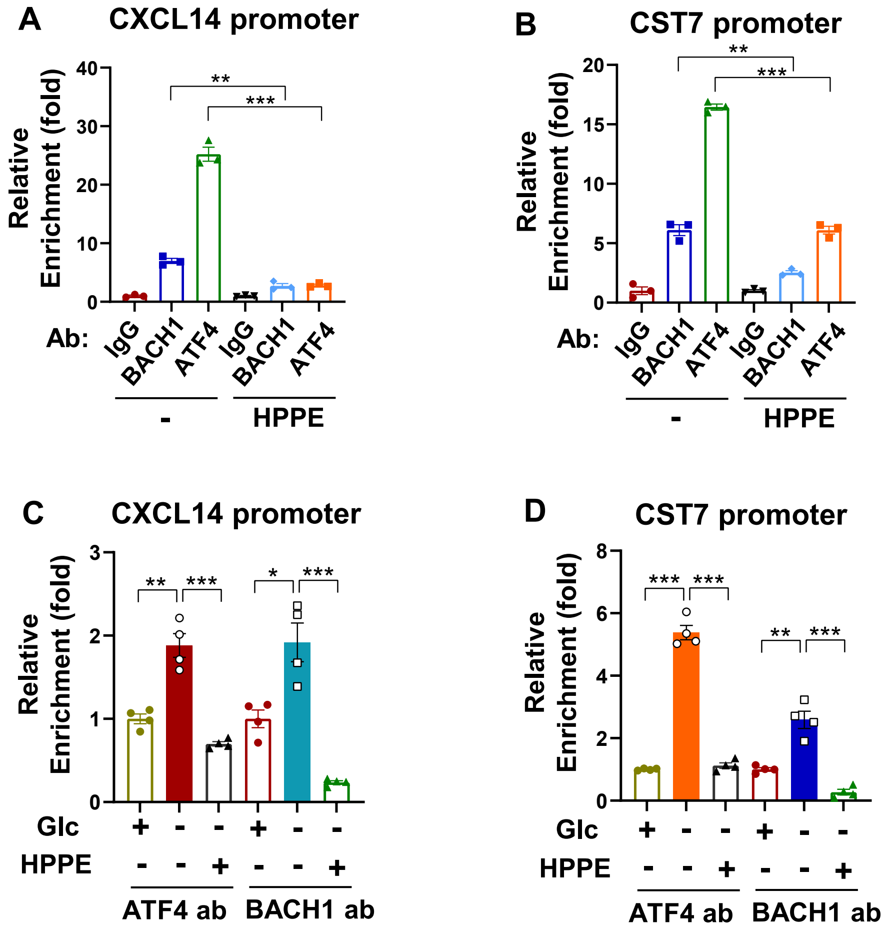

Figure S5. Glucose deprivation-induced ATF4 expression enhances the docking of BACH1 on its pro-metastatic target promoters, related to Figure 7.

(A-B) ChIP analysis of the recruitment of BACH1 or ATF4 to the promoters of CXCL14 (A) and CST7 (B) in MDA-MB-231 cells treated with/without 2.5 μM HPPE (n=3).

(C-D) ChIP analysis in MDA-MB-231 cells under glucose starvation and non-starvation conditions, examining the recruitment of BACH1 or ATF4 to the promoters of CXCL14 (C) and CST7 (D), in the presence or absence of HPPE (n=4). Glc (glucose), 4.5g/L; HPPE, 2.5 μM.

In (A-D), data represent mean ± SEM; n=3 or 4 technical replicates; p values were calculated using unpaired two-tailed Student's t test. *$p < 0.05$, **$p < 0.01$, ***$p < 0.001$.

Figure S6

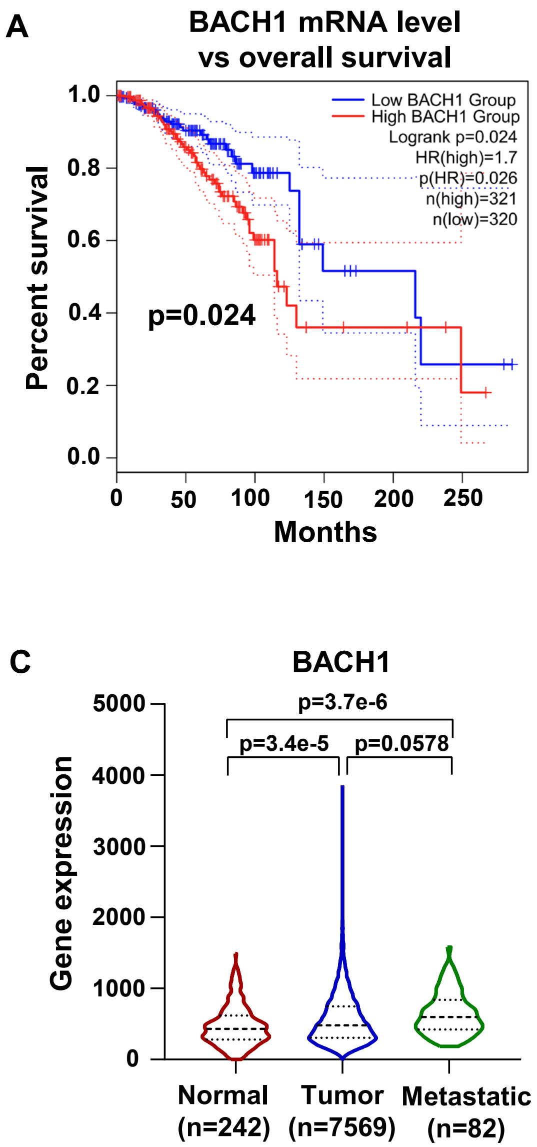

Figure S6. Clinical implications.

(A-B) Correlation between the mRNA expression of BACH1 (A) and CEMIP (B) and the overall survival of patients with invasive breast carcinoma. For both BACH1 and CEMIP, the cutoff for the high-expression group is 70%, and the cutoff for the low-expression group is 30%. n (BACH1 high) = 321, n (BACH1 low) = 320; n (CEMIP high) = 321, n (CEMIP low) = 320. Data were obtained from the database GEPIA2.

(C) Differential BACH1 expression levels in normal breast tissues (n=242), breast tumors (n=7569), and metastatic tumors (n=82) using gene chip data from the TNMplot database.

In (A-B), p values were calculated by log-rank test. In (C), p values were calculated using unpaired two-tailed Student's t test.

Figure S7

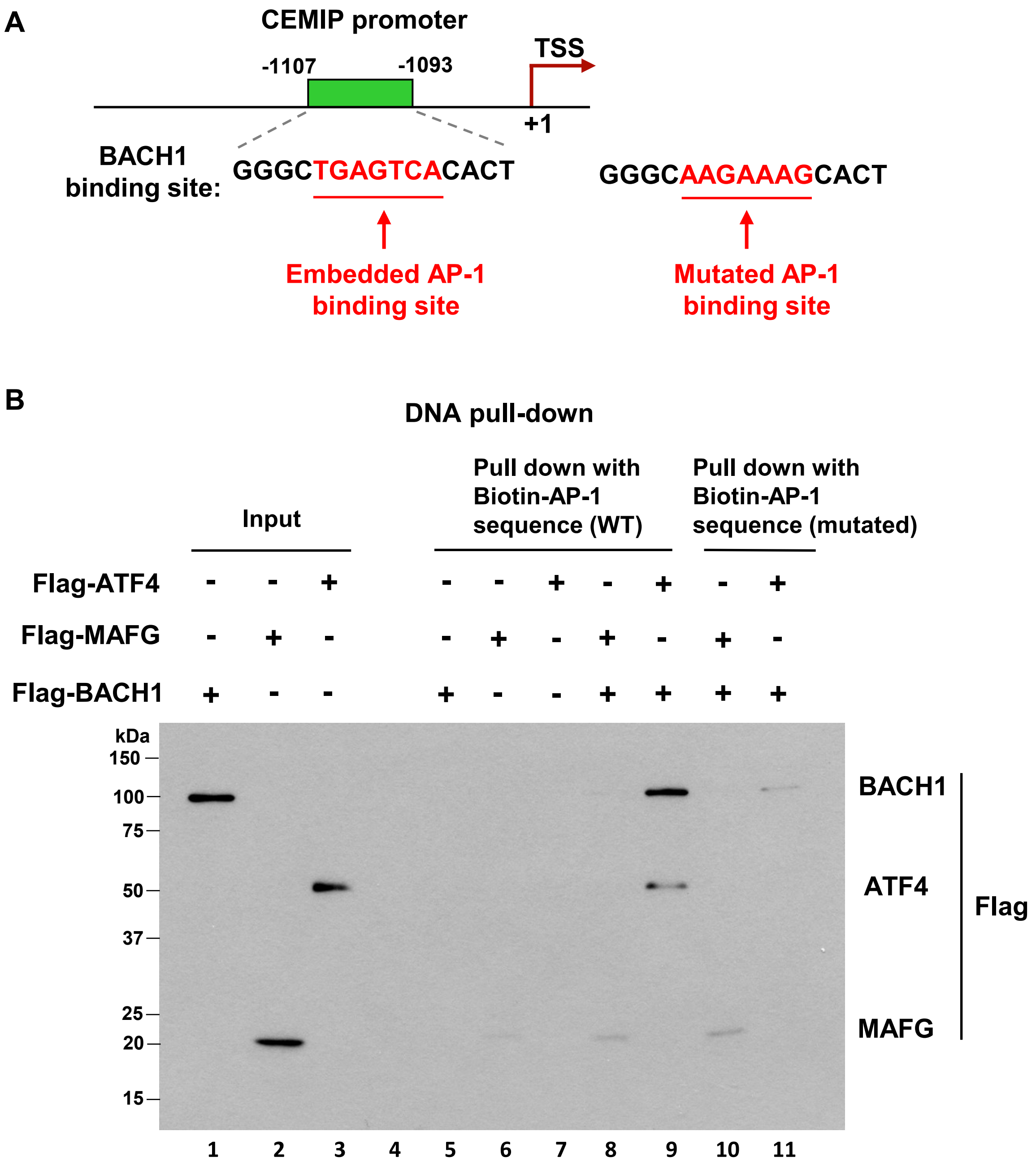

Figure S7. DNA pull down assay for binding of AP-1 related sequence with BACH1-MAFG or BACH1-ATF4.

(A) Diagram of BACH1 binding site and predicted AP-1 binding site on CEMIP promoter. An AP-1 binding site (-1103 to -1097) was predicted in CEMIP promoter by JASPAR (https://jaspar2020.genereg.net/).

(B) DNA pull-down assay. DNA oligos (~70bp) containing wildtype AP-1 binding site (TGAGTCA) or mutated AP-1 binding site (AAGAAAG) were conjugated with Biotin and bound to streptavidin beads, then incubated with purified Flag tagged BACH1, MAFG, ATF4, BACH1+MAFG, and BACH1+ATF4 proteins O/N. Binding proteins were eluted with Biotin and detected by Western blot using Flag antibody.

Figure S8

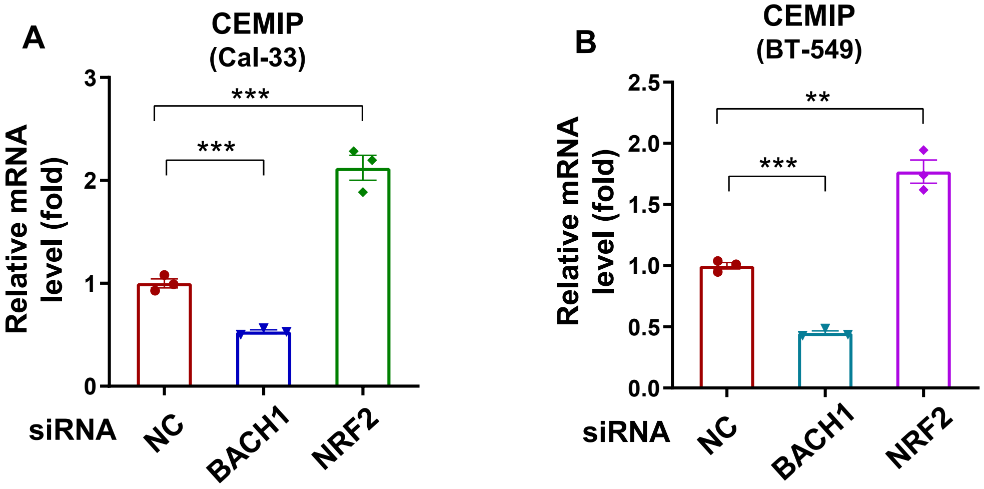

Figure S8. The regulation of NRF2 on CEMIP expression.

(A-B) qPCR analysis of CEMIP mRNA levels in Cal-33 cells (A) and BT-549 cells (B) after transfection with BACH1 or NRF2 siRNA for 48h.

In (A-B), data represent mean ± SEM; n=3 technical replicates; p values were calculated using unpaired two-tailed Student's t test. **$p < 0.01$, ***$p < 0.001$.

Table S1. Diet Formulas

Diets used in this study were from Teklad Diets, Inotiv

Control Diet (93M) (TD.150345)

Formula	g/kg
Casein	100
DL-Methionine	1.6
Corn Starch	512.46
Sucrose	100
Maltodextrin	155
Vegetable Shortening, hydrogenated (Crisco)	25
Corn Oil	25
Cellulose	34.918
Vitamin Mix, AIN-93-VX w/ Cellulose (110068)	15
Thiamin (81%)	0.01
Vitamin K1, phylloquinone	0.002
Choline Bitartrate	2.5
Mineral Mix, w/o Ca & P (98057)	13.39
Calcium Phosphate, dibasic	9.8
Calcium Carbonate	5.25
TBHQ, antioxidant	0.07

Selected Nutrient information:

	% by weight	% by kcal from
Protein	8.9	9.7
Carbohydrate	70.8	77.7
Fat	5.1	12.6
Kcal/g	3.6	

Ketogenic 93M Diet (TD.160153)

Formula	g/kg
Casein	180
DL-Methionine	2.88
Vegetable Shortening, hydrogenated (Crisco)	440
Cocoa Butter	150
Corn Oil	85
Cellulose	59.1884
Vitamin Mix, AIN-93-VX w/ Cellulose (110068)	27
Thiamin (81%)	0.018
Vitamin K1, phylloquinone	0.0036
Choline Bitartrate	4.5
Mineral Mix, w/o Ca & P (98057)	24.1
Calcium Phosphate, dibasic	17.64
Calcium Carbonate	9.54
TBHQ, antioxidant	0.13

Selected Nutrient information:

	% by weight	% by kcal from
Protein	15.9	9.5
Carbohydrate	0	0
Fat	67.7	90.5
Kcal/g	6.7	

Table S2. Antibodies used in this paper

Name	Company	Catalog and RRID
BACH1 (clone F9) (WB, 1:1000)	Santa Cruz Biotechnology	Cat#sc-271211; RRID:AB_10608972
BACH1 (ChIP, 2-4μg/ml cell lysate)	R and D Systems	Cat#AF5776; RRID:AB_2061974
ATF4 (clone D4B8) (WB, 1:1000; IP, 1:50; ChIP, 1:50; IHC, 1:50)	Cell Signaling	Cat#11815; RRID:AB_2616025
ATF4 (clone B3) (WB, 1:500)	Santa Cruz	Cat#sc-390063; RRID:AB_2810998
Flag (clone M1) (WB, 1:1000)	Sigma	Cat#F3040; RRID:AB_439712
Vinculin (clone hVIN-1) (WB, 1:3000)	Sigma	Cat#V9131; RRID:AB_477629
SLC7A11 (clone D2M7A) (WB, 1:1000)	Cell Signaling	Cat#12691; RRID:AB_2687474
CEMIP (WB, 1:3000, suitable for human)	Novus Biologicals	Cat#45750002; RRID:AB_2234168
CEMIP (clone E4JIZ) (WB, 1:1000, suitable for human and mouse)	Cell Signaling	Cat#72197
β-actin (WB, 1:2000)	Abcam	Cat#ab8227; RRID:AB_2305186
PUMA (clone D30C10) (WB, 1:1000)	Cell Signaling	Cat#12450; RRID:AB_2797920
Normal rabbit IgG (IP/ChIP, 2-4 μg/ml lysate)	Santa Cruz	Cat#sc-2027; RRID:AB_737197
Normal goat IgG (ChIP, 2-4 μg/ml lysate)	R&D	Cat#AB-108-C; RRID:AB_354267
HRP-conjugated anti-mouse (WB, 1:10000)	SouthernBiotech	Cat#1031-05; RRID:AB_2794307
HRP-conjugated anti-rabbit (WB, 1:10000)	SouthernBiotech	Cat#4050-05; RRID:AB_2795955

Table S3. Primers, siRNAs, and gRNAs used in this paper

**qPCR primers**		
Human SLC7A11	TCTCCAAAGGAGGTTACCTGC	AGACTCCCCTCAGTAAAGTGAC
Human GAPDH	CCCATGTTCGTCATGGGTGT	TGGTCATGAGTCCTTCCACGATA
Human CEMIP	GAACCCGGCACATCCTGATT	GATCCGGCTGAATACCTTCATC
Human BACH1	ATGTCTCTGAGTGAGAACTC	TTACTCATCAGTAGTACATTTATC
Human CXCL14	CATCACCACCAAGAGCGTGT	TTCTCGTTCCAGGCGTTGTA
Human CST7	GTGTGAAGCCAGGATTTCCTAA	TGTCGTTCGTGCAGTTGTTGA
Human NGFR	CCTACGGCTACTACCAGGATG	CACACGGTGTTCTGCTTGT
Human PGF	GACGTTCTCTCAGCACGTTCG	AACAGCATCGCCGCACAG
Human ATF4	CCCTTCACCTTCTTACAACCTC	TGCCCAGCTCTAAACTAAAGGA
Human NRF2	TCCAGTCAGAAACCAGTGGAT	GAATGTCTGCGCCAAAAGCTG
Human β-actin	AGAGGGAAATCGTGCGTGAC	CAATAGTGATGACCTGGCCGT
Mouse CEMIP	TGGCAGGAGCCACTACTACTG	CCCTTTCGTTCTGGGCTTTTAAT
Mouse NGFR	CCTGGACAGTGTTACGTTCTC	ACACAGGGAGCGGACATACT
Mouse CST7	GCCGAACTACATGCAGGAAGA	GGCAGGGTTGGTTTGGAAGT
Mouse CXCL14	AGTGTAAGTGTTCCCGGAAGG	GCAGTGTGGGTACTTTGGCTT
Mouse β-actin	AGAGGGAAATCGTGCGTGAC	CAATAGTGATGACCTGGCCGT
**ChIP qPCR primers (human)**		
CEMIP	GCGAAAGTGTGGGCAGTAA	ACTCAGTGATGAACGGGGC
CXCL14	CACCAGAGGGCAGCAAAGAG	TTGTTGCCGTCCTTATGCAG
CST7	GCGGTTGCTTTGTGGCTGAT	GTGTTTTGCTGCTGAATCATTTTGG
NGFR	AAGCAGTCCCTGTCTGGAAG	GGCTGGACATAAGAGGCCAG
**siRNAs and Crispr gRNAs**		
ON-TARGETplus human ATF4 siRNA, SMARTpool	Cat#L-005125-00-0020	Horizon Discovery
ON-TARGETplus human BACH1 siRNA, SMARTpool	Cat#L-007750-00-0020	Horizon Discovery
ON-TARGETplus non-targeting pool	Cat#D-001810-10	Horizon Discovery
ON-TARGETplus human NRF2 (NFE2L2) siRNA, SMARTpool	Cat#L-003755-00-0005	Horizon Discovery
BACH1 TrueGuide™ Synthetic sgRNA-1	Cat#A35533; ID: CRISPR792568_SGM	Thermo Fisher Scientific
BACH1 TrueGuide™ Synthetic sgRNA-2 (custom)	Cat#A35534; sequence: AGAAACGGUCUGAGUGUCCG	Thermo Fisher Scientific
ATF4 TrueGuide™ Synthetic sgRNA	Cat#A35533; ID: CRISPR988840_SGM	Thermo Fisher Scientific

Uncropped Western blot images

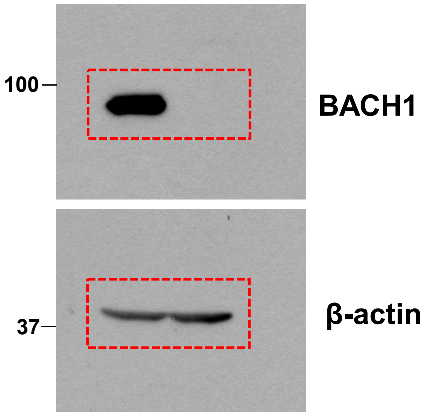

Fig.2B

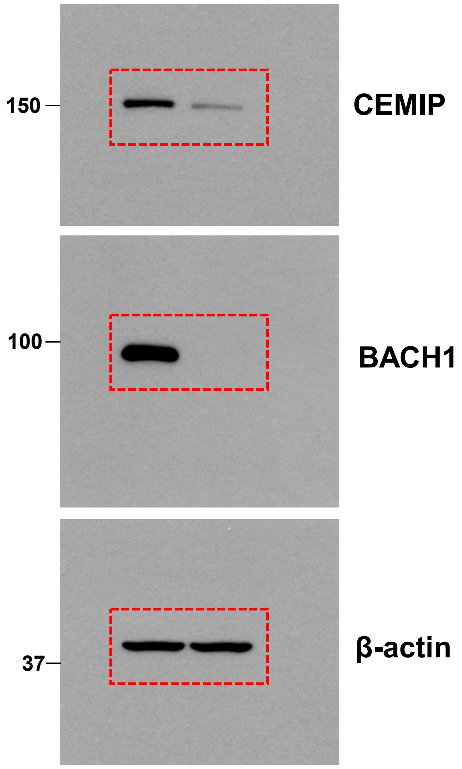

Fig.2D

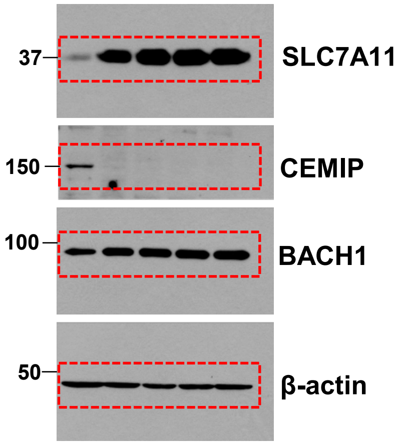

Fig.4K

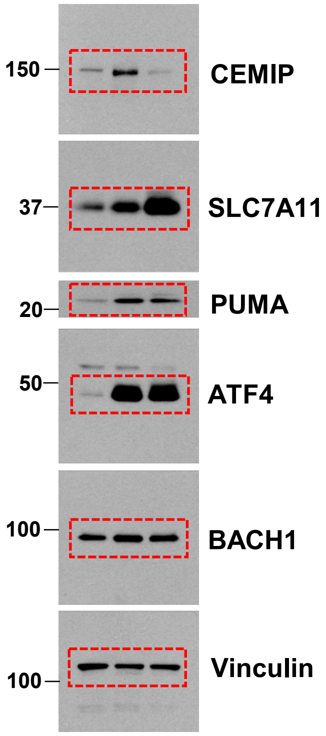

Fig.4L

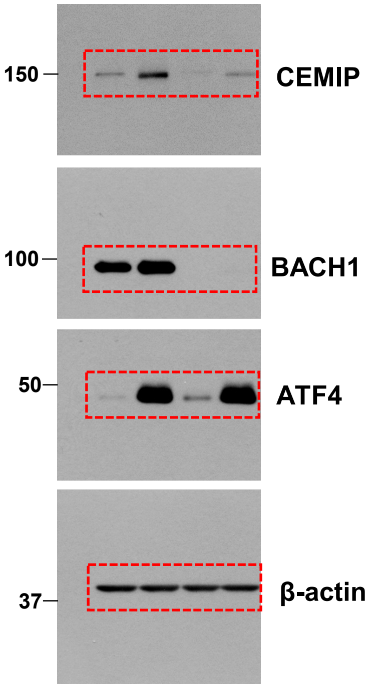

Fig.4M

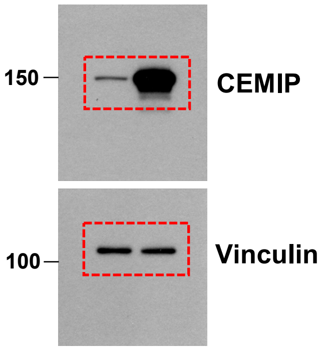

Fig.4N

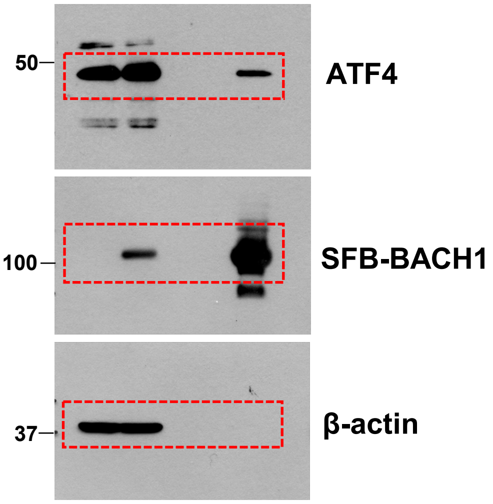

Fig.5A

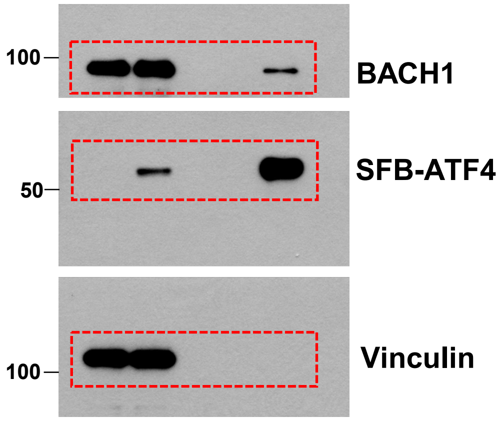

Fig.5B

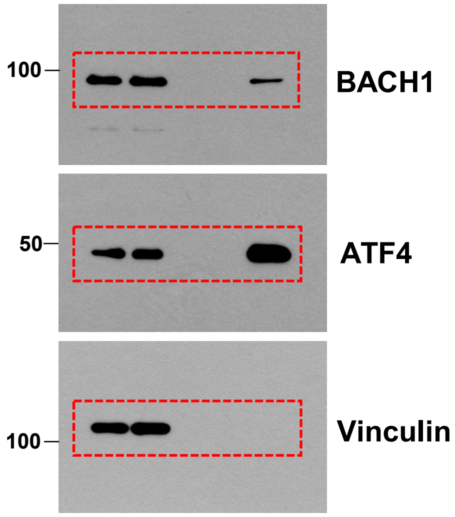

Fig.5C

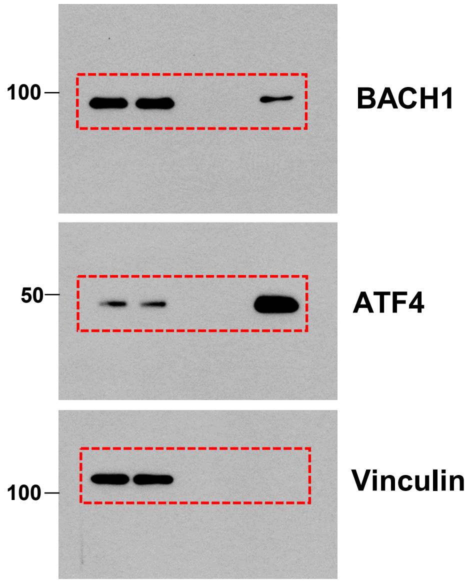

Fig.5D

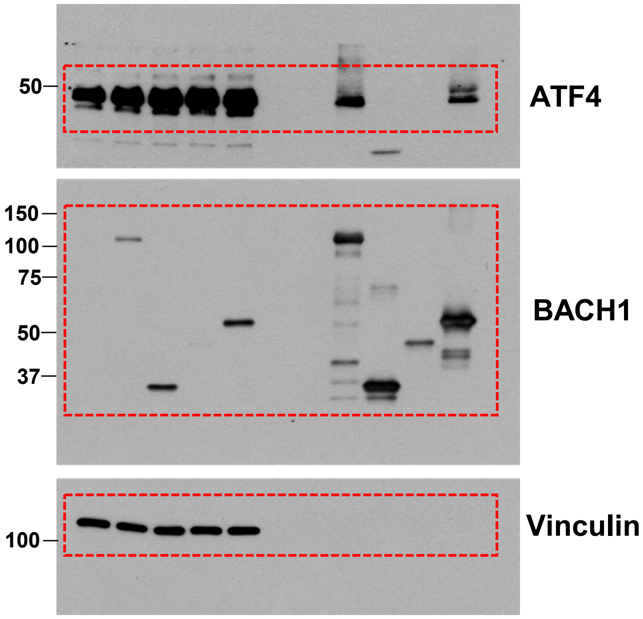

Fig.5E

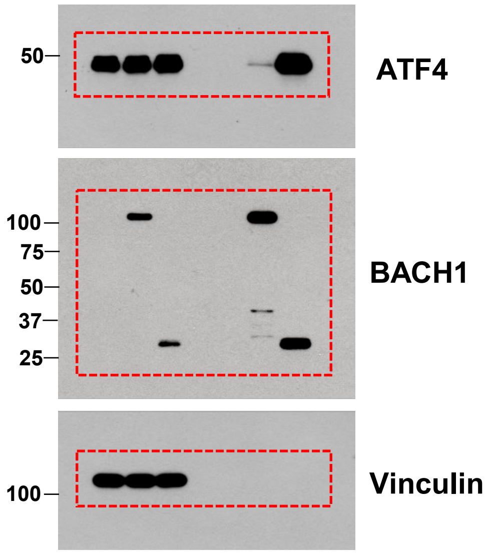

Fig.5F

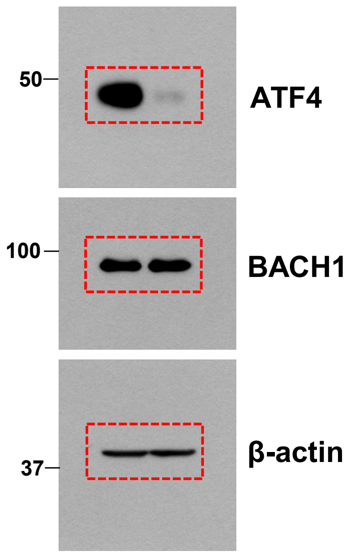

Fig.6I

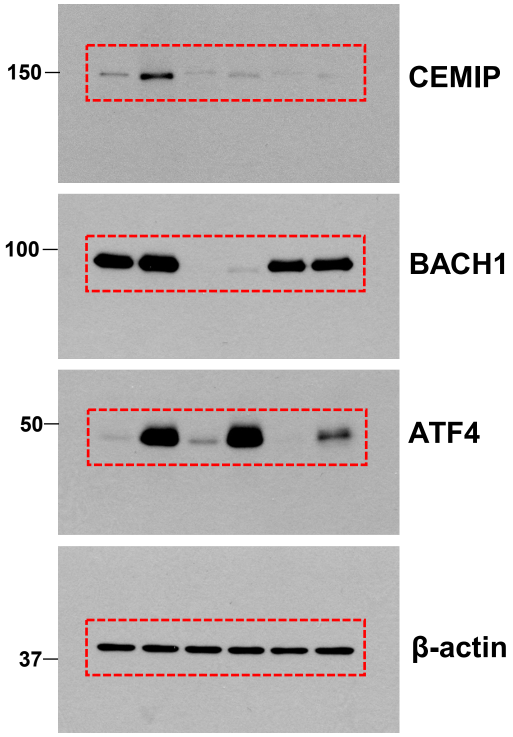

Fig.6J

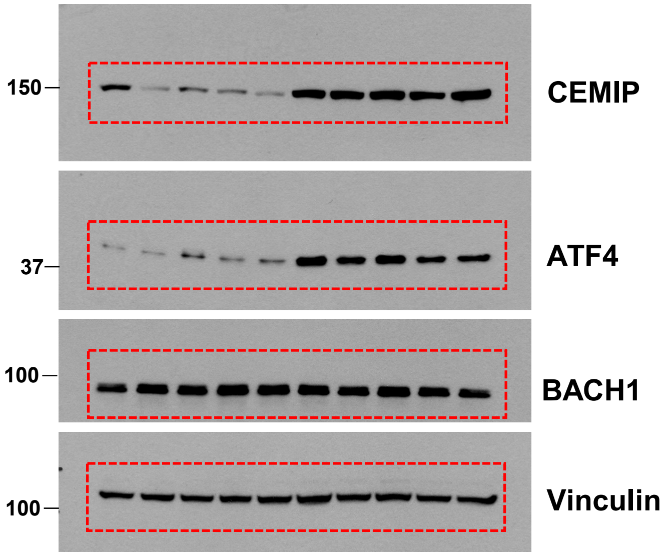

Fig.8E

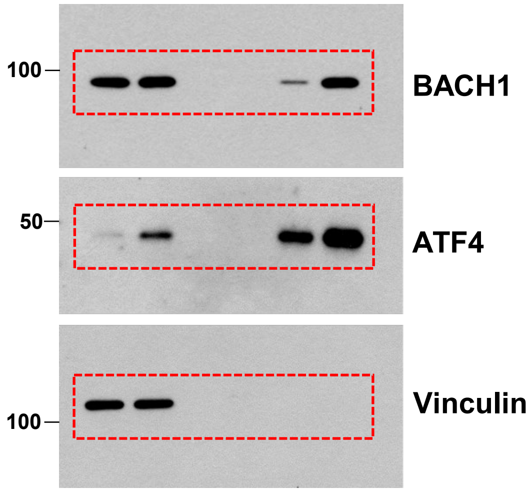

Fig.8F

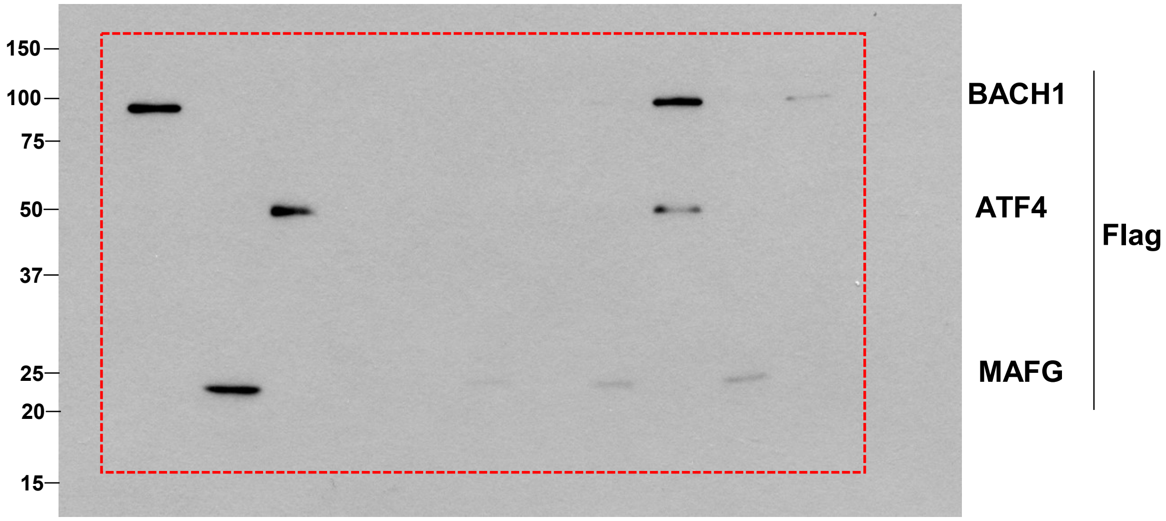

Fig.S7B
